# An opportunistic evaluation of a routine service improvement project to reduce falls in hospital

**DOI:** 10.1186/s12913-021-06073-4

**Published:** 2021-01-22

**Authors:** Diane Sheppard, Elaine Clarke, Karla Hemming, James Martin, Richard Lilford

**Affiliations:** 1 University Hospital Coventry and Warwickshire, Coventry, CV2 2DX UK; 2 Institute of Applied Health Research, University of Birmingham, Edgbaston, B15 2TT UK

**Keywords:** Patient safety, Falls, Time series, Implementation, Rapid response evaluation

## Abstract

**Background:**

Preventing falls in hospital is a perennial patient safety issue. The University Hospital Coventry and Warwickshire initiated a programme to train ward staff in accordance with guidelines. The National Institute for Health Research Collaboration for Leadership in Applied Health Research and Care West Midlands was asked to expedite an independent evaluation of the initiative. We set out to describe the intervention to implement the guidelines and to evaluate it by means of a step-wedge cluster study using routinely collected data.

**Methods:**

The evaluation was set up as a partially randomised, step-wedge cluster study, but roll-out across wards was more rapid than planned. The study was therefore analysed as a time-series. Primary outcome was rate of falls per 1000 Occupied Bed Days (OBDs) collected monthly using routine data. Data was analysed using a mixed-effects Poisson regression model, with a fixed effect for intervention, time and post-intervention time. We allowed for random variations across clusters in initial fall rate, pre-intervention slope and post-intervention slope.

**Results:**

There was an average of 6.62 falls per 1000 OBDs in the control phase, decreasing to an average of 5.89 falls per 1000 OBDs in the period after implementation to the study end. Regression models showed no significant step change in fall rates (IRR: 1.02, 95% CI: 0.92–1.14). However, there was a gradual decrease, of approximately 3%, after the intervention was introduced (IRR: 0.97 per month, 95% CI: 0.95–0.99).

**Conclusion:**

The intervention was associated with a small but statistically significantly improvement in falls rates. Expedited roll-out of an intervention may vitiate a step-wedge cluster design, but the intervention can still be studied using a time-series analysis. Assuming that there is some value in time series analyses, this is better than no evaluation at all. However, care is needed in making causal inferences given the non-experimental nature of the design.

**Supplementary Information:**

The online version contains supplementary material available at 10.1186/s12913-021-06073-4.

## Background

Implementation science reports often start with the development of an intervention through stages, as recommended in the MRC Framework for Complex Interventions [1]. This development pathway encompass literature reviews, theory development, studies of barriers and facilitators, iterative co-production of the intervention (perhaps guided by a framework such as LEAN), and pilot studies. Such a formal and stylised approach has obvious merits, and the academic literature is replete with examples thereof. However, this method is resource-intensive and time-consuming – hospital managers want quick results and have many competing demands on their time. It is therefore interesting and informative to examine the effectiveness of routine service improvement initiatives that are implemented without academic input. Such an opportunity arose recently at the University Hospital Coventry, part of the University Hospitals Coventry and Warwickshire NHS Trust. This is one of the largest teaching Trusts in the UK, operating from two acute general hospitals in the West Midlands, with a total of 1230 beds and 8.405 staff. This study relates to the main hospital site, which has 1100 beds. The in-patient specialties for this hospital are given under study design.

The hospital decided to implement routine guidance from the Royal College of Physicians (RCP) [2] and from the National Institute for Health and Care Excellence (NICE) [3] for the prevention of falls in hospital. Approximately 250,000 falls are recorded per year in English NHS hospitals, with an estimated cost to the NHS of £2.3 billion per year [3]. Falls are the most commonly reported patient safety incident. The national mean rate of falls is 6.6 per 1000 occupied bed days (OBDs), and up to 30 % of falls result in physical injury [4]. It is thus important to reduce the incidence of falls and to do so without limiting people’s independence or freedom of movement.

The Chief Nurse, having decided to implement the intervention across the hospital, requested that the National Institute for Health Research (NIHR) Collaboration for Leadership in Applied Health Research and Care (CLAHRC) West Midlands to conduct an independent evaluation using routinely collected data. The Chief Nurse and other stakeholders agreed to randomise the order of roll-out of the intervention across many (but not all) of the wards, thereby creating a ‘partial’ step-wedge design. However, the researchers played no part in the design or implementation of the intervention, and could not influence the rate of implementation. We thought it would be interesting to evaluate an intervention put in place by the service with no academic input, in contrast to the extensive literature we summarise in the discussion.

## Methods

### Overview

This implementation and evaluation covered all 36 wards in the hospital. The study was set up in such a way as to include 19 of the 36 wards in a step-wedge cluster RCT. The remaining 17 wards were deemed unsuitable for randomisation for reasons given below. Since some wards were included in the step-wedge and others not, we refer to a partial step-wedge design. However, roll-out across all the wards was extremely rapid, effectively obliterating the steps in the step-wedge component. We therefore deviated from the original design and analysed the effect of the intervention over all wards (randomised or not) by means of a standard time series analysis, using the methods and yielding the results reported here.

The goals of research were:

- To describe the intervention designed to implement the RCP and NICE guidelines.
- To evaluate the intervention by analysing routinely collected falls data by means of a step-wedge design.

As we shall describe, the step-wedge design ‘collapsed’ as the roll-out of the intervention was too rapid. We therefore had to modify our goal and analyse data as a time-series analysis. Our study therefore also provides a lesson in one of the difficulties that can be encountered when a research team attempts to analyse ‘real world’ interventions over which it has no control.

### Intervention

We describe the intervention as far as possible according to the TIDieR check list [5].

#### Item 1. Brief intervention name

No name was assigned.

#### Item 2. Rationale and essential elements

The purpose of the intervention was to implement the RCP [2] and NICE [3] guidelines for the reduction of falls. These guidelines were formulated on the basis of evidence on the effectiveness of different actions that might reduce falls without unduly restricting patients’ freedom of movement. The guidelines specify the particular actions that should, or should not, be a part of patient care. The guidelines specify that implementation should be guided by a ‘multi-disciplinary group’ to review falls data and respond accordingly. This function was subsumed in the hospital by an existing ‘falls-forum’. This intervention to implement the guidelines can be conceptualised on three levels:

1. Actions taken at the level of the falls-forum.
2. Actions taken to enhance implementation of the guidelines.
3. Compliance with the guidelines at the level of the ward and individual patients.

**Fig. 1:**
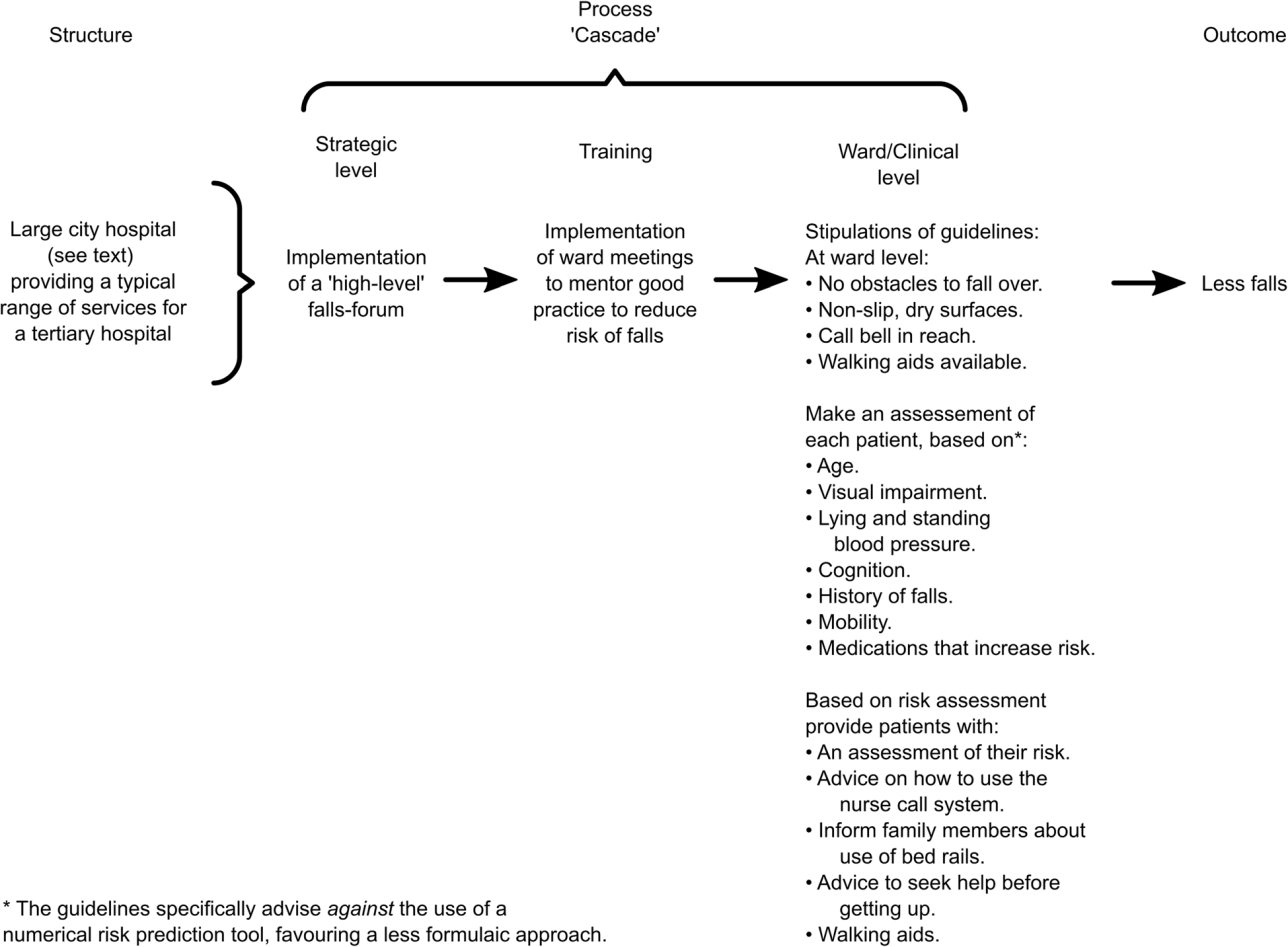
Representation of the cascade of processes through which the intervention was intended to reduce fall rates

This cascade, based on Donabedian’s original framework [6], is represented in slightly modified form [7] in Fig. 1. The individual actions at ward and patient level from the RCP/NICE guidelines are also listed.

#### Item 3. Materials

Training materials were produced under supervision from the falls-forum (level 1). This involved production of a set of instructions to help staff identify patients at greatest risk of falling. This was deemed necessary because the above guidance explicitly proscribes use of a numerical falls prediction tool and it was felt that staff would find alternative materials helpful. The instructions were developed interactively following two pilot implementations. The instructions were incorporated in a booklet on General Risk Assessment, enclosed as Appendix A. The falls-forum also developed the ward-based training intervention.

#### Item 4. Procedures

The falls-forum (level 1 above) met monthly to review falls data and take action accordingly. Training meetings involving groups of wards (level 2 above) were arranged to motivate and train ward staff to comply with the RCP and NICE guidelines and to introduce the booklet. The intervention was designed to enhance awareness of the problem, improve knowledge of methods to reduce falls, develop communities of practice at ward staff level, and to develop familiarity with the above new booklet. Insofar as this would be successful, it would result in improved compliance with the guidelines at ward and patient level (level 3).

#### Item 5. Personnel

The falls-forum is comprised of the Associate Director of Nursing – Patient Safety and Quality, a consultant gerontologist, nursing matrons, the hospital Falls Lead (a physiotherapist), a further physiotherapist, a pharmacist, and a member of the hospital quality team. At the level of enhanced implementation (level 2) the Falls Lead met with Ward Managers to arrange meetings with groups of about ten senior nurses from each ward. Two training meetings were held with each ward, timed to coordinate with ‘safety huddles’ during the morning and evening nursing staff hand-over. For example, staff would be mentored in the meetings to ensure that surfaces were kept dry and that a walking aid was available to patients, as these measures are stipulations of the guidelines (Fig. 1). Likewise, staff were instructed in how they should make an assessment of patient risk, based on visual acuity, history of falls and other risk factors in Fig. 1. Although they made an assessment of risk they did not use a risk score, again in line with the guidelines.

#### Item 6. Modes of delivery

Face-to-face meetings were arranged at both falls-forum and ward levels, involving the staff members mentioned above and the frequencies mentioned below.

#### Item 7. Locations

The falls-forum met at the hospital headquarters. The ward meetings took place in a meeting room at ward level.

#### Item 8. Frequency

**Fig. 2:**
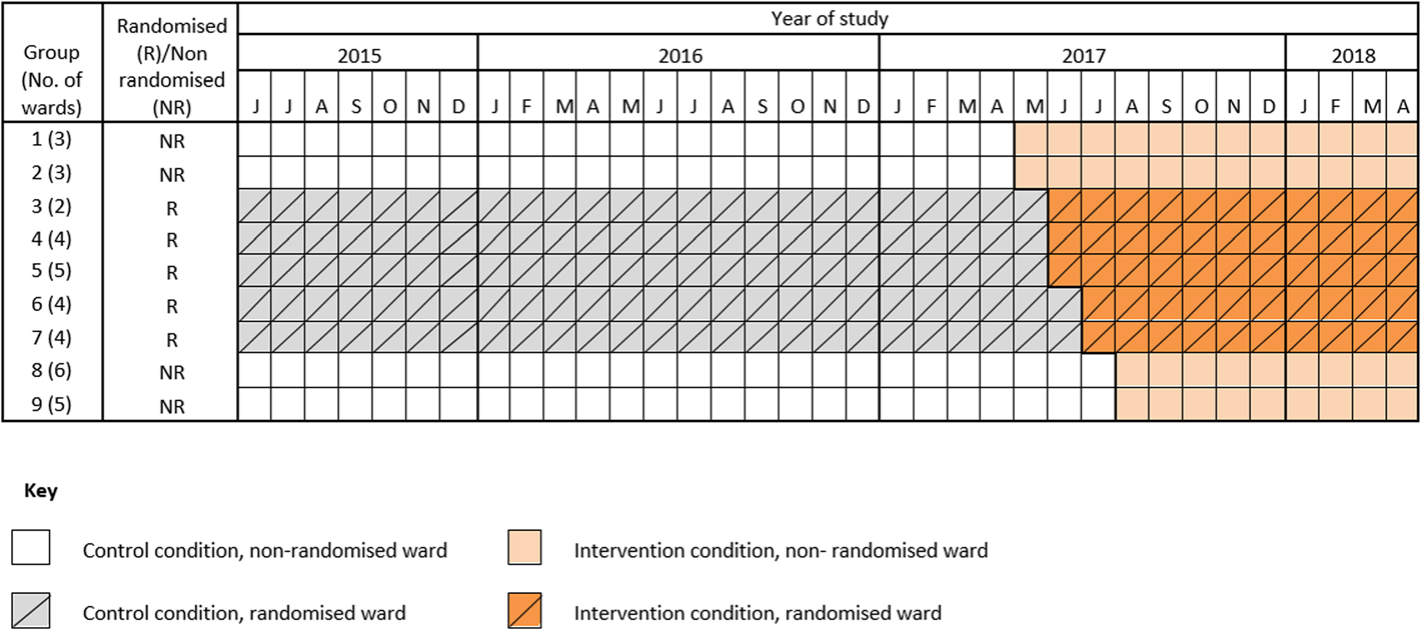
Schematic representation of the rollout of the intervention to randomised and non-randomised clusters. Data was collected each month from June 2015 to April 2018

The falls-forum met monthly, as stated, and provided oversight of the intervention over the intervention period, May 2017 to August 2017 (Fig. 2). The two ward level meetings took place one to two weeks apart, lasting for about one hour.

#### Items 9 and 10. Tailoring or modifications

There was no specific tailoring or modification of the messages as all interventions were applied in acute hospital wards.

#### Items 11 and 12. Implementation fidelity

No formal fidelity measurement was made, but attendance at the teaching meetings was reported to be high and Ward Managers arranged ad hoc training sessions to disseminate the training programme for staff who had been unable to attend the training meetings. The number of, and attendance at, these meetings was not recorded.

### Falls data

Data were available on the rate of falls per 1000 OBDs. The primary outcome for this study was the rate of falls per 1000 OBDs. Within each ward, the senior nurse on duty has an obligation to report all falls into the incident reporting system, Datix, as soon as feasible after the fall. The number of OBDs is recorded routinely by the hospital informatics centre. Falls are recorded on a monthly basis per 1000 OBDs in all wards. We obtained this monthly aggregated data June 2015 to April 2018. Data was collected from all 36 wards within the hospital.

### Study design

The intervention was rolled-out to all 36 wards in the hospital. Wards were arranged in nine groups of between two and six wards (Table 1). The grouping of wards was determined by the Assistant Director of Patient Safety and Quality on the basis of speciality. Four groups (17 wards) were deemed by the Assistant Director of Patient Safety and Quality to be unsuitable for randomisation, two because roll-out was imminent, and two because they were short stay wards. This left five groups (19 wards) that were eligible for randomisation in a step-wedge trial [8]. Groups were randomised to a sequence. A schematic representation of the roll-out is given in Fig. 2. However, the Assistant Director of Patient Safety and Quality perceived an urgency to deliver the intervention as she felt it would be wrong to delay introduction of a service from which she expected patients to benefit. Roll-out was very rapid, to the degree that the intervention was rolled out over the five randomised groups of wards over two months, effectively obliterating the step-wedge (Fig. 2). We therefore deviated from the original plan and analysed the data as a time-series study.

**Table 1:** List of 36 included wards

Ward No	Description	Randomisation Order
40	Gerontology - Age related, Rehab	Not randomised
20	Gerontology	
21	Gerontology	
41	Stroke	Not randomised
42	Neurology	
43	Neurosurgery	
10	Cardiology	1
11	Cardiothoracic Surgery	
Cedar	Orthopaedics area	2
Hoskyn		
Mulberry		
Oak	Rehab area	
32	Head & Neck	3
33^a^	Surgery	
33	Gastro	
33	Urology	
33	Short Stay	
30	Respiratory	4
31	Medical ward	
34	Clinical Haematology	
35	Oncology	
50	Renal	5
52	Orthopaedics	
53	Orthopaedics	
53 MTEC	Major Trauma Care	
21^a^	Short Stay	Not randomised
21	General Surgery	
22^a^	ECU	
22	Surgical Assessment Unit	
22A	Vascular Surgery	
23	Gynaecology Suite	
12/CDU	Acute Medical Unit 1	Not randomised
3	Rheumatology / Medicine	
12	Acute Medical Unit 3	
1	Observation / Assessment Unit (ED)	
AMU 2	Acute Medical Unit 2	

^a^ Some of the above wards are sub-divided into different areas. These wards are re-labelled sequentially as ‘clusters’ 1 to 36 for the analysis

### Statistical analysis

The primary outcome for the study was rate of falls per 1000 OBDs. The primary analysis was conducted using segmented regression. To this end, a mixed-effects Poisson regression model was fitted to the data. A fixed effect was included for intervention condition, time, and time since intervention (interaction between time and intervention arm). This model assumes a linear trend in the outcome prior to the intervention, a shift in the outcome at the time of the intervention, and then a new linear trend in the outcome post-intervention. A random effect was included for cluster (ward), time and post-intervention time. It is likely that the wide variation in falls rates pre-intervention between clusters/wards was related partly to differences in case mix. Consequently random effects were added to allow for random variations across clusters for: the baseline rate of falls (so at June 2015 each cluster has a different baseline value for the rate of falls); the effect of time pre intervention (so each cluster follows a different linear trend pre-intervention); and the effect of time post-intervention (and each cluster follows a different linear trend post intervention). Our objective was to fit a model with an additional random effect component for the intervention (to allow the intervention effect as characterised by its shift to the system, to vary across clusters) but models with this random effect failed to converge (could not be fitted by software programme). This means that we cannot examine for heterogeneous treatment effects. Data were checked for auto-correlation in each cluster, using partial auto-correlation plots (See Appendix B, Fig. S1). No evidence of auto correlation was found.

As a sensitivity analysis, a model with a cubic spline for time pre-intervention and a second cubic spline for time post-intervention was fitted to the data. This model allows for a non-linear trend for time that can differ pre- and post-intervention. To explore differential intervention effects across wards, we fitted to each cluster independently, a Poisson regression model with a fixed effect for intervention condition, time, and time since intervention (interaction between time and intervention arm). This model assumes a linear trend in the outcome prior to the intervention, a shift in the outcome at the time of the intervention, and then a new linear trend in the outcome post-intervention. This allowed the change in time trend, and the immediate intervention effect to vary across clusters. Two wards were excluded from this sub-analysis of differential treatment effects as they had no observations contributing to the post-intervention period.

### Trial registration

This study was registered as a step-wedge cluster trial on ClinicalTrials.gov NCT 03192384 (20/06/2017).

## Results

All occupied bed days and all falls between June 2015 and April 2018 contributed to the study. The intervention was rolled out between May 2017 and August 2017. The randomised rollout of the intervention occurred in June and July 2017.

### Primary analysis

**Fig. 3:**
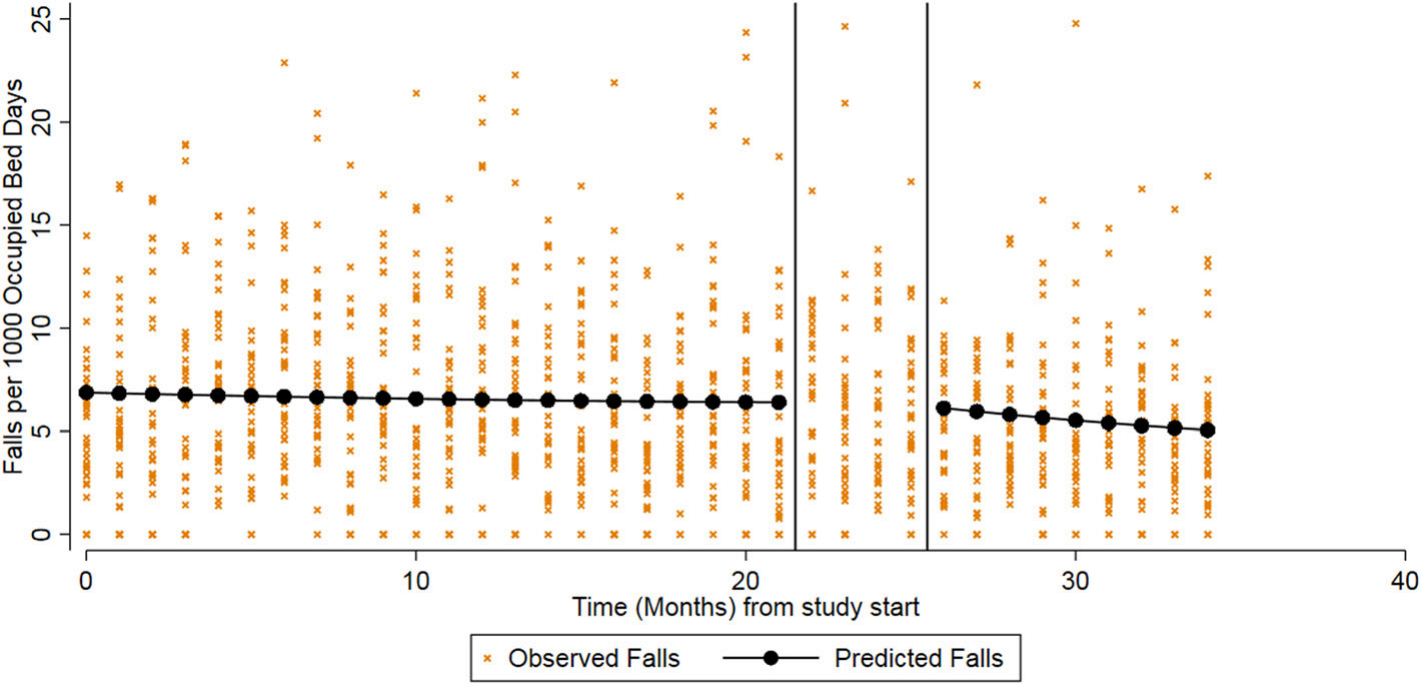
Observed vs predicted falls per 1000 bed days using an interrupted time series analysis with segmented regression. The black vertical lines indicate the beginning and end of the intervention implementation

The results for the primary analysis are given in Table 2, and the model is illustrated in Fig. 3. There was an average of 6.62 falls per 1000 OBDs recorded during the control period. This decreased during the post-intervention period, to an average of 5.89 per 1000 OBDs. In June 2015 (the first month of the study), the rate of falls was 6.54 per 1000 OBDs (95% CI: 5.76 to 7.43). During the control phase (June 2015 to May 2017) there was a slight decrease in falls per month, though this was not statistically significant (IRR: 0.99, 95%CI: 0.99 to 1.00). The point estimate here is indicating that every month the rate of falls is decreasing by 1% compared to the previous month.

**Table 2:** Impact of the intervention on the rate of falls

Average number of falls per 1000 bed days over study period		Initial rate of falls (Intercept)		Time (Pre-intervention slope)		Intervention (Shift)		Post intervention time (Post-intervention slope)	
Control, mean (SD)	Intervention, mean (SD)	IR (95% CI)	*p*-value	IRR^*^ (95% CI)	*p*-value	IRR^**b**^ (95% CI)	*p*-value	IRR^a^ (95% CI)	*p*-value
6.62 (4.80)	5.89 (5.38)	6.54 (5.76–7.43)	< 0.001	0.99 (0.99–1.00)	0.087	1.02 (0.92–1.14)	0.669	0.97 (0.95–0.99)	< 0.001

*IR* Incidence rate, *IRR* Incidence rate ratio, *CI* Confidence interval

^a^ The estimate here indicates the change each month in the rate of falls compared to the previous month.

^b^ The estimate here indicates the change in the rate of falls in the month immediately following the intervention implementation to the month preceding it.

When the intervention was implemented, there was no evidence of an immediate or step change in the rate of falls: the IRR shows a slight, but non-significant increase in rate of falls of 2% in the month immediately following the intervention implementation (IRR 1.02, 95% CI: 0.92 to 1.14).

Following roll-out of the intervention, there is a significant decrease in the rate of falls (IRR 0.97, 95% CI: 0.95 to 0.99): that is to say, every month the rate of falls decreases by 3% compared to the previous month.

Results were robust to the sensitivity analysis for non-linear time trends (Appendix B, Tables S1 and S2, and Fig. S2).

**Fig. 4:**
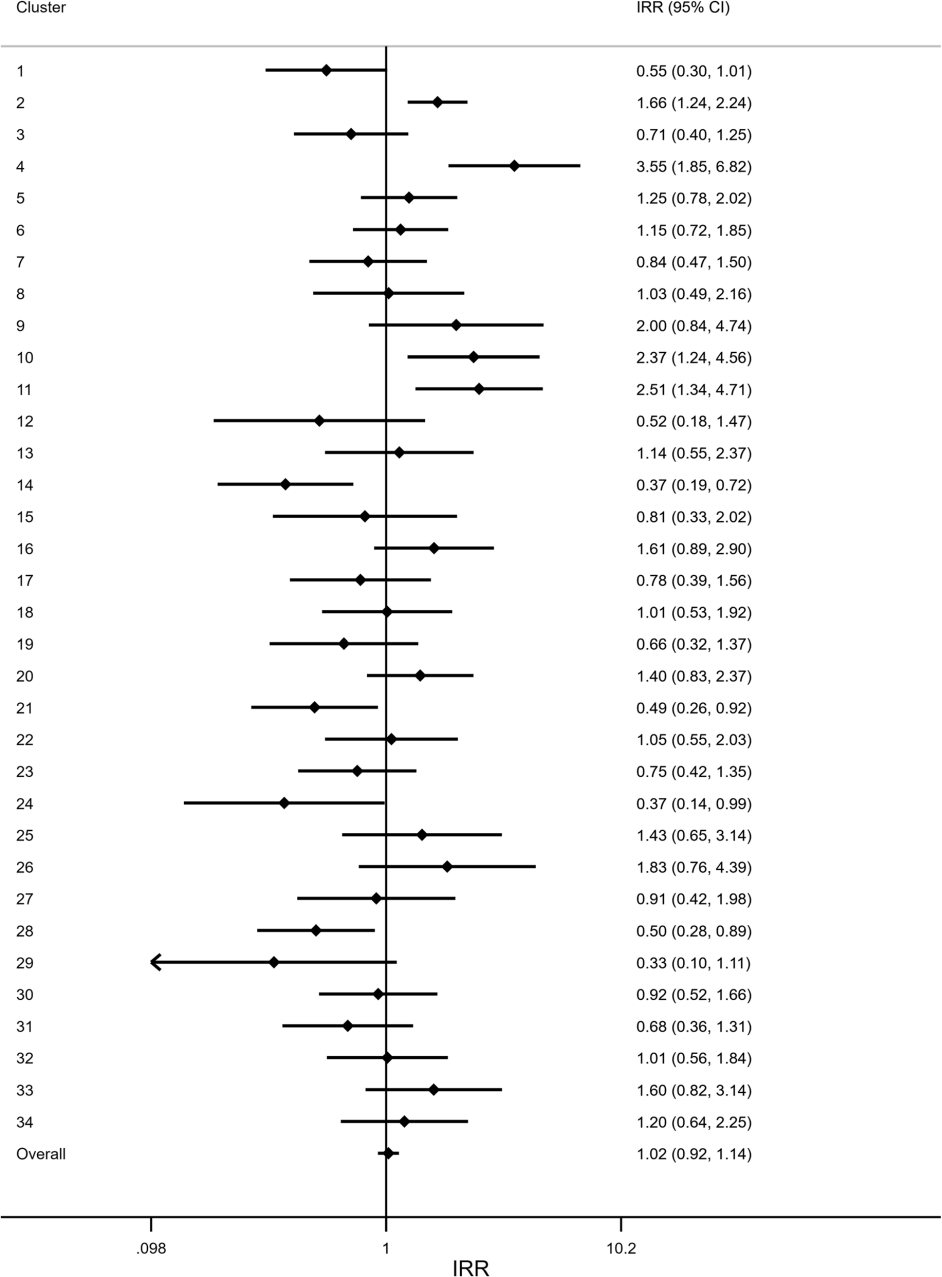
Forest plot of the immediate intervention effect in each ward. To explore differential intervention effects across wards, a Poisson regression model was fitted to each ward individually. The estimate here indicates the change in the rate of falls in the month immediately following the intervention implementation to the month preceding it. The overall estimate was obtained by fitting a mixed-effect Poisson regression model to the entire dataset. This model included a fixed effect for: intervention, time, and a time by intervention interaction; and random effects for: ward, time, and post-intervention time. IRR: Incidence rate ratio. CI: Confidence interval

The sensitivity analysis identified a large degree of heterogeneity across wards in the effect of the intervention (Fig. 4). Although, overall, there was an increase in falls in the first post-intervention month (as previously stated), some wards showed evidence of a positive effect, whilst others displayed evidence of a negative effect. Cluster 14 (Ward 33, Urology), for example, showed a 63% reduction in falls in the month following the intervention (IRR: 0.37, 95% CI: 0.19 to 0.72). In contrast, Cluster 4 (Ward 41, Stroke) found a sharp increase in rate of falls (IRR: 3.55, 95% CI: 1.85 to 6.82) (Fig. 4).

**Fig. 5:**
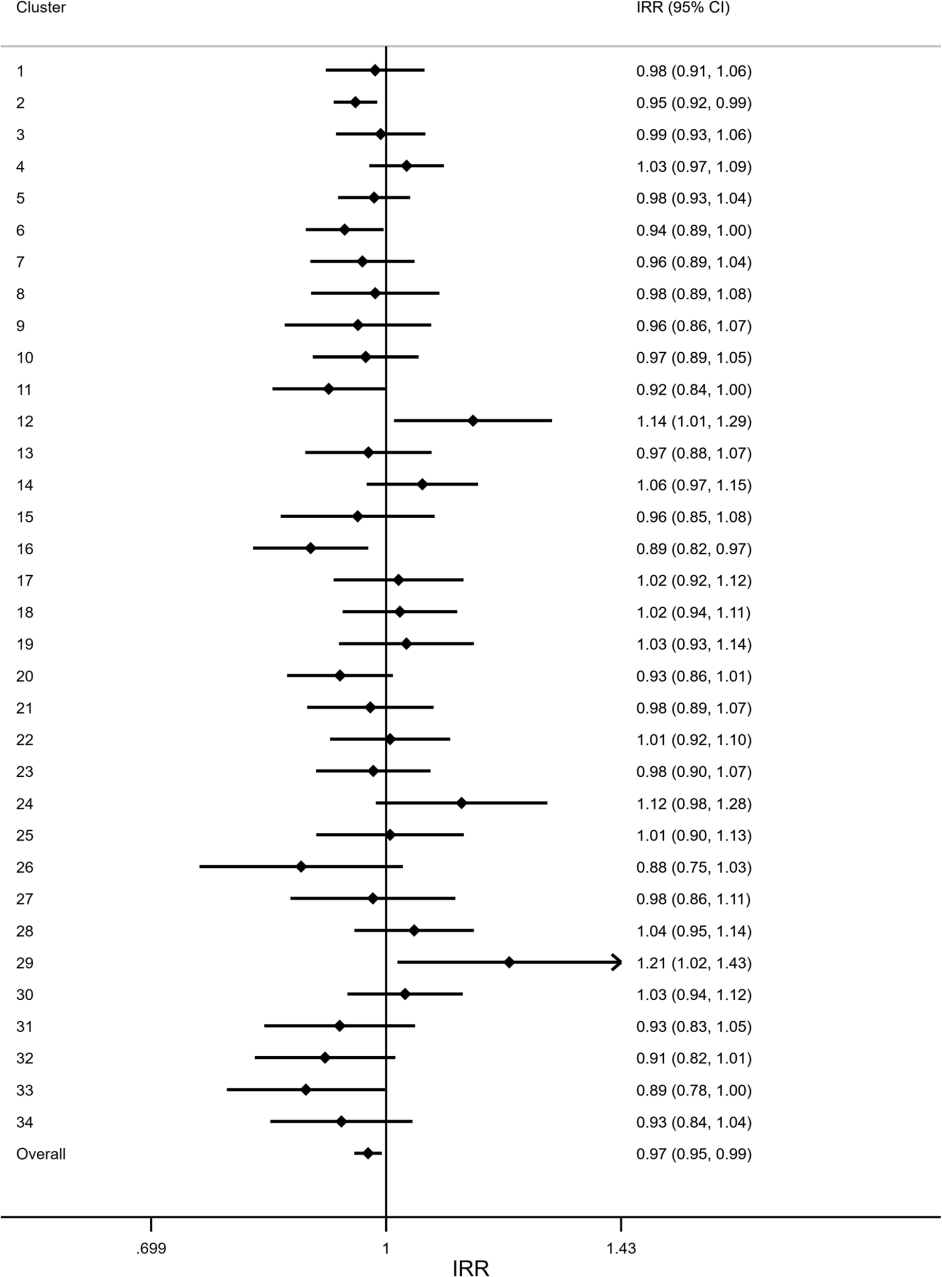
Forest plot of the post-intervention time trend in each ward. To explore differential intervention effects across wards, a Poisson regression model was fitted to each ward individually. The estimate here indicates the post-intervention change each month in the rate of falls compared to the previous month. The overall estimate was obtained by fitting a mixed-effect Poisson regression model to the entire dataset. This model included a fixed effect for: intervention, time, and a time by intervention interaction; and random effects for: ward, time, and post-intervention time. IRR: Incidence rate ratio. CI: Confidence interval

In addition to the heterogeneity in the immediate effect of the intervention, there was also a large degree of heterogeneity across wards in the post-intervention time trend in each ward (Fig. 5). Some wards showed evidence of a decreasing trend in falls over time, for example Cluster 16 (Ward 30, Respiratory) showed a 11% reduction in falls per month following the intervention (IRR: 0.89, 95% CI: 0.82 to 0.97). However, some wards, showed an increase in falls per month post-intervention, such as Cluster 12 (Ward 33, Surgery), which showed a 14% increase in falls per month (IRR: 1.14, 95% CI: 1.01 to 1.29).

## Discussion

### Headline findings

The rate of falls prior to intervention was similar to the national average and was declining gradually in the hospital. This study has shown that implementation of an intervention based on national guidelines was associated with a subsequent increase in the rate at which falls declined (averaged across the whole hospital). However, we identified no clear ‘step change’ immediately following the intervention, suggesting that it may have taken time for the intervention to be assimilated and ‘bed-down’ in practice. We also identified considerable variation in both the immediate and long-term effects of the intervention – suggesting possible variation in the fidelity with which staff translated learning from ward sessions into practice.

### Comparison with other studies

The intervention was designed to train/educate staff to follow the guidelines for fall prevention. There is considerable literature on the prevention of falls in general, and in hospital in particular [9–13]. Miake-Lye and colleagues provide a thorough account of falls prevention in hospital [14]. This review builds on reviews by the Cochrane Collaboration [13], Oliver [9], and Cousement [15]. They included numerous clinical processes that should be followed on the wards to reduce falls. No one study included all of these, but the practice recommended in the guidelines (ward/patient level in Fig. 1) were all included in the list. The Miake-Lye overview also included eleven studies that looked specifically at implementation – the topic of this paper. Eight of these mentioned top leadership support, such as in our study, and all but one mentioned multi-disciplinary teams, as reported here (and recommend by NICE). Five of the eleven studies mentioned making a special effort to engage (rather than just mentor/train) front-line staff, although it is difficult to quantify or verify the extent to which this happens. Training was mentioned in two studies, but the dose (two one-hour sessions in our study) was seldom given. Nine studies mentioned piloting or an incremental approach, and this was lacking in our study – indeed it was rolled out over four months. Observations of the attitudes of staff was mentioned in eight studies, but this was not attempted in our study since we operated in ‘rapid response’ mode, and did not have time to solicit funds for a detailed qualitative study or any other aspect of implementation fidelity.

The Miake-Lye study cites a meta-analysis of 12 studies showing an 18% relative risk reduction in falls of borderline significant (95% CI 0.68–1.00). Many reviewers hesitate to conduct such quantitative syntheses, given not only differences in context, but in the interventions themselves. The effect we observed was modest, but at 3% per month, it is not out of line with the summary statistics in the literature. Moreover, even modest effects such as those observed here, are worthwhile if an intervention is inexpensive [7]. It is also noteworthy that the slope of the improvement continued until the end of the study and it would be interesting to track falls rates over a longer period using the methods proposed here. It is possible that improvements in outcome become harder to achieve (and measure) as the base-rate declines. It is also likely, or at least it may be hypothesised, that a more intensive intervention (more than two training sessions of one-hour each) would have yielded a larger effect. This might have provided more time for staff engagement.

### Limitations

We had planned a step-wedge randomised design. This would have been a stronger basis for cause and effect inferences. The time series used in this study has strengths compared to a single before-and-after measurement. Moreover, a long run of pre-intervention data can detect a series of bad results that can prompt an intervention and create a false impression of effectiveness as the data ‘regress to the mean’. This was not the case here. Nevertheless, this is a study with no contemporaneous, let alone randomised, controls. Ultimately the reader must make a judgement – perhaps assisted by counterfactual reasoning [16]. This study is based on routinely reported data and it is known that such data underestimates the ‘true’ falls rate [10]. A bias would arise if reporting was ‘reactive’ meaning that the extent of underreporting interacted with intervention status. Such a possibility cannot be excluded, even in a study with contemporaneous controls. Given greater resources, it would have been possible to implement a second set of observations with independent observers. However, the continued improvement over many months does not suggest that reporting bias was a problem in this study, on the assumption that it is unlikely that staff across a large hospital could titrate reporting fidelity to yield the picture of gradual improvement observed.

### Rapid response studies

As the rollout was more rapid than originally proposed, we were not able to evaluate the intervention as intended – i.e. by partial cluster randomised controlled step-wedge trial. The advantages of performing a stepped-wedge trial were heavily attenuated, and an interrupted time series with segmented regression was the only viable option. This study is an example of a ‘rapid response’ study, where the nature and time-table for deployment are under the control of the service and the evaluation may have to be adapted accordingly [17].

This study was set up as the evaluation of a ‘routine’ improvement method in a busy hospital, rather than an ‘implementation science project’. While in one sense our study describes one intervention to address one problem in just one place, our purpose in publishing this study is to provide an example of the evaluation of an intervention totally designed and implemented by the hospital. We also document one of the difficulties that rapid response research may present. When implementation follows a service, rather than a scientific imperative, protocols may need to be adapted. In this case an attempt at a step-wedge design was vitiated by rapid roll-out of the implementation.

Reference to the board papers of any healthcare institution will quickly show that the great majority of service intervention across the globe follow the ‘in service, by service, for service’ model, rather than an implementation science approach co-produced with external input, following the MRC guidance for complex interventions [1], and the full panoply of EQUATOR recommendations [18]. We therefore wish to encourage independent evaluations of routine service interventions, which we think should be more widely used. We plan to continue this time series and track the effect of further improvement initiatives to reduce falls. We argue that such evaluations of routine service-based initiatives should complement, but certainly not replace, more formal co-production implementation science projects. In particular, we argue that principles established in ‘implementation science evaluations’ should be widely disseminated to inform routine service initiatives. Moreover, as an implementation science project is gradually assimilated and incorporated into routine practice, evaluations of roll-out can assess the generalisability/ transferability of formal implementation science findings

## Conclusion

We have described an intervention at general hospital and ward level to implement guidelines to reduce falls in hospital. We attempted to evaluate this intervention through a step-wedge cluster trial. Roll-out was faster than anticipated and we therefore conducted an interrupted time-series study based on routinely collected data. Our findings suggest that an intervention based on established principles may have resulted in a reduction in falls rates in a large city hospital. The magnitude of any effect was modest, but reductions in falls rates continued over many months. We think that more extensive use should be made of independent assessments of quality and safety interventions.

## Supplementary Information

**Additional file 1: Appendix A.** Patient Risk Assessment Booklet. An example of the patient risk assessment booklet used in the study to help staff identify patients at greatest risk of falling.

**Additional file 2: Appendix B.** Supplementary tables and figures.

## Data Availability

The data that supports the findings of this study are routine aggregated data, which are the property of University Hospitals Coventry and Warwickshire NHS Trust, and so are not publicly available. Any request to obtain or analyse the data would need to be made to University Hospitals Coventry and Warwickshire NHS Trust.

## Abbreviations

CI: Confidence Interval
CLAHRC: Collaboration for Leadership in Applied Health Research and Care
IRR: Incidence Rate Ratios
NICE: National Institute for Health and Care Excellence
NIHR: National Institute for Health Research
OBD: Occupied Bed Days
RCP: Royal College of Physicians

## References

[1] Craig P, Dieppe P, Macintyre S, Michie S, Nazareth I, Petticrew M (2008). Developing and evaluating complex interventions: the new Medical Research Council guidance. BMJ..

[2] Royal College of Physicians (2017). National Audit of Inpatient Falls. Audit report 2017.

[3] National Institute for Health and Care Excellence (2013). Falls in older people: assessing risk and prevention. CG161.

[4] Healey F, Scobie S, Oliver D, Pryce A, Thomson R, Glampson B (2008). Falls in English and welsh hospitals: a national observational study based on retrospective analysis of 12 months of patient safety incident reports. Quality Saf Health Care.

[5] Hoffmann TC, Glasziou PP, Boutron I, Milne R, Perera R, Moher D (2014). Better reporting of interventions: template for intervention description and replication (TIDieR) checklist and guide. BMJ..

[6] Donabedian A (1980). Explorations in quality assessment and monitoring.

[7] Lilford RJ, Chilton PJ, Hemming K, Girling AJ, Taylor CA, Barach P (2010). Evaluating policy and service interventions: framework to guide selection and interpretation of study end points. BMJ..

[8] Hemming K, Lilford R, Girling AJ (2015). Stepped-wedge cluster randomised controlled trials: a generic framework including parallel and multiple-level designs. Stat Med.

[9] Oliver D, Healey F, Haines TP (2010). Preventing falls and fall-related injuries in hospitals. Clin Geriatr Med.

[10] Hill AM, McPhail SM, Waldron N, Etherton-Beer C, Ingram K, Flicker L (2015). Fall rates in hospital rehabilitation units after individualised patient and staff education programmes: a pragmatic, stepped-wedge, cluster-randomised controlled trial. Lancet..

[11] Haines TP, Hill AM, Hill KD, Brauer SG, Hoffmann T, Etherton-Beer C (2013). Cost effectiveness of patient education for the prevention of falls in hospital: economic evaluation from a randomized controlled trial. BMC Med.

[12] Haines TP, Bennell KL, Osborne RH, Hill KD (2004). Effectiveness of targeted falls prevention programme in subacute hospital setting: randomised controlled trial. BMJ..

[13] Cameron ID, Dyer SM, Panagoda CE, Murray GR, Hill KD, Cumming RG (2018). Interventions for preventing falls in older people in care facilities and hospitals. Cochrane Database Syst Rev.

[14] Miake-Lye IM, Hempel S, Ganz DA, Shekelle PG. Chapter 19. Preventing In-Facility Falls. In: Shekelle PG, Wachter RM, Pronovost PJ, et al., editors. Making health care safer II: an updated critical analysis of the evidence for patient safety practices. 13-E001-EF. Rockville (MD): Agency for Healthcare Research and Quality (US); 2013. p. 178–200.

[15] Coussement J, De Paepe L, Schwendimann R, Denhaerynck K, Dejaeger E, Milisen K (2008). Interventions for preventing falls in acute- and chronic-care hospitals: a systematic review and meta-analysis. J Am Geriatr Soc.

[16] Pearl J, Glymour M, Jewell NP (2016). Causal inference in statistics: a primer.

[17] Lilford R (2019). Demand-led research: a taxonomy. NIHR CLAHRC West Midlands News Blog.

[18] EQUATOR Network. Enhancing the QUAlity and transparency of health research 2019 Available from: https://www.equator-network.org/reporting-guidelines/. [Last Accessed 26 Nov 2020].

