# Supplementary material for: An opportunistic evaluation of a routine service improvement project to reduce falls in hospital
Source: BMC Health Serv Res. 2021 Jan 22;21:79. doi: 10.1186/s12913-021-06073-4 (PMC7821491; doi:10.1186/s12913-021-06073-4)
Supplement: Supplementary file 1 — Additional file 1: Appendix A. Patient Risk Assessment Booklet. An example of the patient risk assessment booklet used in the study to help staff identify patients at greatest risk of falling. [file 12913_2021_6073_MOESM1_ESM.pdf]

NHS
University Hospitals
Coventry and Warwickshire
NHS Trust

FOR SCANNING PURPOSES
AFFIX PATIENT LABEL

Patient Risk Assessment Booklet

Re-assessment to take place weekly as a minimum or as frequently as condition dictates

If patient is transferred ensure assessments are reviewed

Please use appropriate care plans associated with risk assessments as indicated

These are located via the Trust intranet
Click ***DIRECTORATES AND DEPARTMENTS***, then select ***PRACTICE DEVELOPMENT***, then ***RECORD KEEPING AND DOCUMENTATION***
Further copies of each risk assessment are also available here

We **Care.** We **Achieve.** We **Innovate.**

Risk Assessment Number 1
INFECTION

Is the patient presenting with any of the following. Tick YES or NO and follow the actions	NO	YES	Tick Actions taken
**AN ALERT ON CRRS?**<br><br>**MRSA**---- Commence MRSA quick action guide			Quick action guide commenced ☐<br>Patient Isolated ☐<br>Full MRSA Screen Taken ☐<br>Decolonisation commenced ☐
**ESBL/Gentamycin Resistant Organism**---<br>Manage as positive for duration of stay			Quick action guide commenced ☐<br>Patient Isolated ☐
**MRAB-Multi Resistant AcinetoBacter**-isolate and contact infection control IMMEDIATELY or medical microbiologist on call.			Quick Action guide commenced ☐<br>Patient Isolated ☐<br>MRAB Screen taken ☐<br>Infection control or microbiology informed ☐
**CDT (*C. difficile*)**—if symptomatic and *C. difficile* suspected start C. *difficile* quick action guide.<br>Please refer to reverse of quick action guide if patient relapses.			Quick action guide commenced ☐<br>Patient Isolated ☐<br>Stool specimen sent ☐
**MDRTB**--- Place in monitored negative pressure room until been assessed by respiratory physician and inform infection control.			Quick action guide commenced ☐<br>Patient Isolated in negative Pressure room ☐<br>Respiratory Physician contacted ☐<br>Infection control or microbiology informed ☐<br>P3 Respirators to be worn ☐
**DIARRHOEA AND/OR VOMITING**<br>If infective cause suspected isolate patient and send stool sample			Isolate patient/s ☐<br>Stool specimen sent for MC&S & verbally inform laboratory of MC&S request ☐<br>Bristol Stool Chart ☐<br>Fluid balance chart ☐
**A RASH of unknown origin**<br>If contagious cause suspected (including not fully dried chicken pox / shingles lesions) isolate patient.			Patient isolated ☐<br>Referred to consultant dermatologist ☐<br>Avoid pregnant staff entering room ☐
**TRANSFERRED FROM ANOTHER HOSPITAL OR REPATRIATED FROM ABROAD**			Patient isolated ☐<br>MRSA screen taken (For UK transfers) ☐<br>MRSA, ESBL & MRAB screen taken (for abroad transfers) ☐
**RETURNED FROM ABROAD WITH A FEVER (within the past 28 days). Excluding North America, Europe, New Zealand, Australia**			Patient isolated ☐
**RESPIRATORY SYMPTOMS**<br>A) Are there features to suggest the possibility of Tuberculosis i.e? Cough > 3weeks, Weight loss, Night sweats. Investigate appropriately and where clinically strongly suspected isolate pending investigations.<br>B) Suspicion of influenza			Patient Isolated in negative Pressure room ☐<br>Respiratory Physician contacted ☐<br>Staff to wear PPE-P3 respirator ☐

**MRSA RISK ASSESSMENT FOR ISOLATION**<br>**To be undertaken only when side rooms are NOT available**	**HIGH RISK of TRANSMISSION / ANTIBIOTIC RESISTANCE**<br>******ISOLATION NECESSARY*******<br>• Deep exuding wounds ☐<br>• Gentamicin / Mupirocin Resistant MRSA ☐<br>• Multiple wounds / pressure ulcers ☐<br>• Dermatitis (ie eczema/psoriasis or any dry scaly skin (patient likely to be heavy disperser of skin cells) ☐<br>• Sputum positive for MRSA ☐<br>• Urinary catheter in situ ☐<br>**if ANY of the above apply patient MUST remain in isolation**
	**LOW RISK of TRANSMISSION**<br>****If isolation room not available, may be nursed on open ward with strict standard precautions**<br>•None of the above risk factors ☐<br>• No skin breaks or a maximum one or two superficial wounds,

CPE (Carbapenemase producing enterobacteraecia) (Tick applicable boxes)

Has the patient been an inpatient in hospital outside of West Midlands or abroad in the past 12 months? ☐

Has the patient been informed that they have been previously positive? ☐

Does the patient have close contact with anyone known to be previously positive? ☐

IF YOU HAVE ANSWERED YES TO ANY OF THESE QUESTIONS THE PATIENT MUST BE SCREENED FOR CPE

- Rectal swab (must have visible faecal matter) **or** Stool specimen
- Isolate and contact Infection Prevention and Control Team or Microbiologist on-call immediately

Date specimens sent...

Name of Assessor:	Signature:	Date:

Appendix A

Risk Assessment Number 9
ORAL ASSESSMENT

Review frequency of assessment if patients condition changes — Date →							
Signature							
Is there any indication of current infection ☐	Y/N	Y/N	Y/N	Y/N	Y/N	Y/N	Y/N
Does the patient require assistance circle ☐	Y/N	Y/N	Y/N	Y/N	Y/N	Y/N	Y/N
if No to both questions above you do not need to undertake full assessment at this time- however re-assess weekly or if condition changes							
**Mouth Opening**							
No Restrictions	1	1	1	1	1	1	1
Restricted Due To Swelling	2	2	2	2	2	2	2
Fixation In Place	3	3	3	3	3	3	3
**Swallowing**							
No Difficulties In Swallowing	1	1	1	1	1	1	1
Some Swallowing Difficulties	2	2	2	2	2	2	2
Unable To Swallow	3	3	3	3	3	3	3
**Ability To Rinse**							
Rinse/Expel Normally	1	1	1	1	1	1	1
Difficulties To Rinse/Expel	2	2	2	2	2	2	2
Unable To Rinse/Expel	3	3	3	3	3	3	3
**Lips**							
Smooth, Pink, Moist	1	1	1	1	1	1	1
Dry, Cracked	2	2	2	2	2	2	2
Ulcerated/Bleeding	3	3	3	3	3	3	3
**Tongue**							
Pink, Moist, Papillae	1	1	1	1	1	1	1
Coated/Loss Of Papillae	2	2	2	2	2	2	2
Blistered/Cracked	3	3	3	3	3	3	3
**Saliva**							
Watery	1	1	1	1	1	1	1
Thick	2	2	2	2	2	2	2
Absent	3	3	3	3	3	3	3
**Mucous Membrane**							
Pink And Moist	1	1	1	1	1	1	1
Reddened/Coated	2	2	2	2	2	2	2
Ulcerated/Bleeding	3	3	3	3	3	3	3
**Teeth/Dentures**							
Clean, No Debris	1	1	1	1	1	1	1
Localised Plaque/Debris	2	2	2	2	2	2	2
Generalised Plaque/Debris	3	3	3	3	3	3	3
Ill Fitting Dentures/Broken Teeth	4	4	4	4	4	4	4
**Pain**							
Pain Free	1	1	1	1	1	1	1
Intermittent Pain	2	2	2	2	2	2	2
Uncontrolled Pain	3	3	3	3	3	3	3
**Mental Status**							
Alert	1	1	1	1	1	1	1
Apathetic/Sedated	2	2	2	2	2	2	2
Uncooperative/Unconscious	3	3	3	3	3	3	3
**Total Score**							
**Signature of Assessing Nurse**							

<11 Low Risk	11-14 Medium Risk	15-22 High Risk
Continue with basic oral care Review weekly	Monitor- report abnormality- continue with basic oral care consider advanced mouth care plan- Review daily	Monitor- report abnormality advanced care plan Review daily

Please use appropriate care plans as indicated-these are located via the Trust intranet on the front page left column Click 'CLINICAL SUPPORT', then select 'NURSING DOCUMENTATION'

Risk Assessment Number 2
PRESSURE ULCERS

FOR SCANNING PURPOSES AFFIX PATIENT LABEL

ALWAYS ACT UPON THE RISK ASSESSMENT SCORE AND IMPLEMENT PREVENTATIVE MEASURES	Date →							
	Time →							
**AGE**	<65	0	0	0	0	0	0	0
	65-80	1	1	1	1	1	1	1
	80+	3	3	3	3	3	3	3
**BUILD/WEIGHT FOR HEIGHT**	(Use BMI Chart)							
	Average	0	0	0	0	0	0	0
	Above average	1	1	1	1	1	1	1
	Below average	2	2	2	2	2	2	2
**NUTRITIONAL/HYDRATION STATUS** (Make one selection only)	Normal appetite/ intake<br>Full enteral/ parenteral nutrition	0	0	0	0	0	0	0
	Reduced appetite /insufficient to maintain weight/ eating ½ meals offered.	2	2	2	2	2	2	2
	Clear fluids/ IVI only	3	3	3	3	3	3	3
	Refusing/ Very poor intake/ Unable to take diet/ NBM	4	4	4	4	4	4	4
**CONTINENCE** (More than one selection should be made)	Complete/catheterised	0	0	0	0	0	0	0
	Occasionally incontinent	1	1	1	1	1	1	1
	Always incontinent	2	2	2	2	2	2	2
	Only incontinent urine	1	1	1	1	1	1	1
	Only incontinent faeces	2	2	2	2	2	2	2
	Doubly incontinent	3	3	3	3	3	3	3
**MOBILITY/MOVEMENT** (Make one selection only)	Independent/ Fully mobile	0	0	0	0	0	0	0
	Assistance needed to move / mobilise	3	3	3	3	3	3	3
	Highly dependent/immobile	5	5	5	5	5	5	5
**FRICTION/SHEAR** (More than one selection can be made)	Not applicable	0	0	0	0	0	0	0
	Poor balance/posture in bed and/or in the chair	1	1	1	1	1	1	1
	Restless/ Fidgety/ Apathetic	1	1	1	1	1	1	1
**PRESSURE** (Make one selection only)	Not applicable	0	0	0	0	0	0	0
	Bedfast (spends the majority of the time in bed)	3	3	3	3	3	3	3
	Chairbound (sits in a chair most of day and/or night)	5	5	5	5	5	5	5
**SKIN CONDITION** (At Risk Areas) (More than one selection can be made)	Healthy	0	0	0	0	0	0	0
	Dry/dehydrated/Tissue paper	1	1	1	1	1	1	1
	Clammy	1	1	1	1	1	1	1
	Oedematous	1	1	1	1	1	1	1
	Discoloured (non blanching erythema/Grade 1 pressure ulcer)	2	2	2	2	2	2	2
	Pressure Ulcer (Grade 2-4) Specify Grade [ ] (complete wound assessment chart)	4	4	4	4	4	4	4
**PREDISPOSING DISEASE** (Make one selection only) e.g. conditions affecting tissue perfusion, Cardiac, renal, respiratory, peripheral vascular disease etc.	None	0	0	0	0	0	0	0
	Chronic stable	3	3	3	3	3	3	3
	Acute/ chronic unstable	6	6	6	6	6	6	6
	Terminal /End of life	8	8	8	8	8	8	8
**PREDISPOSING CONDITION** (More than one selection can be made)	Not applicable	0	0	0	0	0	0	0
	Anaemia Hb<10	1	1	1	1	1	1	1
	Smoking	1	1	1	1	1	1	1
**NEUROLOGICAL DEFICIT** (Make one selection only) e.g. conditions causing a sensory deficit & reducing awareness of potential tissue damage occurring. e.g. diabetes, CVA, MS, confusion, reduced consciousness etc.	None	0	0	0	0	0	0	0
	Chronic stable/controlled/mild	4	4	4	4	4	4	4
	Acute/ chronic unstable/ uncontrolled /severe	6	6	6	6	6	6	6
**PAIN ON MOVEMENT** (Make one selection only)	None	0	0	0	0	0	0	0
	Mild	1	1	1	1	1	1	1
	Moderate	2	2	2	2	2	2	2
	Severe	3	3	3	3	3	3	3
**MEDICATION** (Make one selection only)	Not applicable	0	0	0	0	0	0	0
	High dose steroids, sedatives, long-term use anti-inflammatory drugs, inotropes above 10 mic/min	4	4	4	4	4	4	4
**SURGERY/TRAUMA** (More than one selection can be made)	Not applicable	0	0	0	0	0	0	0
	Trauma to below waist or spine affecting mobility in last 48 hours	5	5	5	5	5	5	5
	Surgery on operating table >2hours in last 48 hours	5	5	5	5	5	5	5
For any patient with a UHCW NHS Trust pressure ulcer assessment of 10 & above complete & implement a preventative care plan as per trust guidelines	**Total Score**							
	**Initials of assessor**							

Re-assess patient AS A MINIMUM WITHIN 24 HOURS OF THE FOLLOWING:- condition/dependency changes, patient is transferred to another ward or hospital, or clinical intervention such as surgery. Adapt care plan according to any changes of score

10-14 At Risk	15-20 Medium Risk	21 and above High Risk

Appendix A

Risk Assessment Number 3
SKIN ASSESSMENT

Mark and number each area of damaged skin

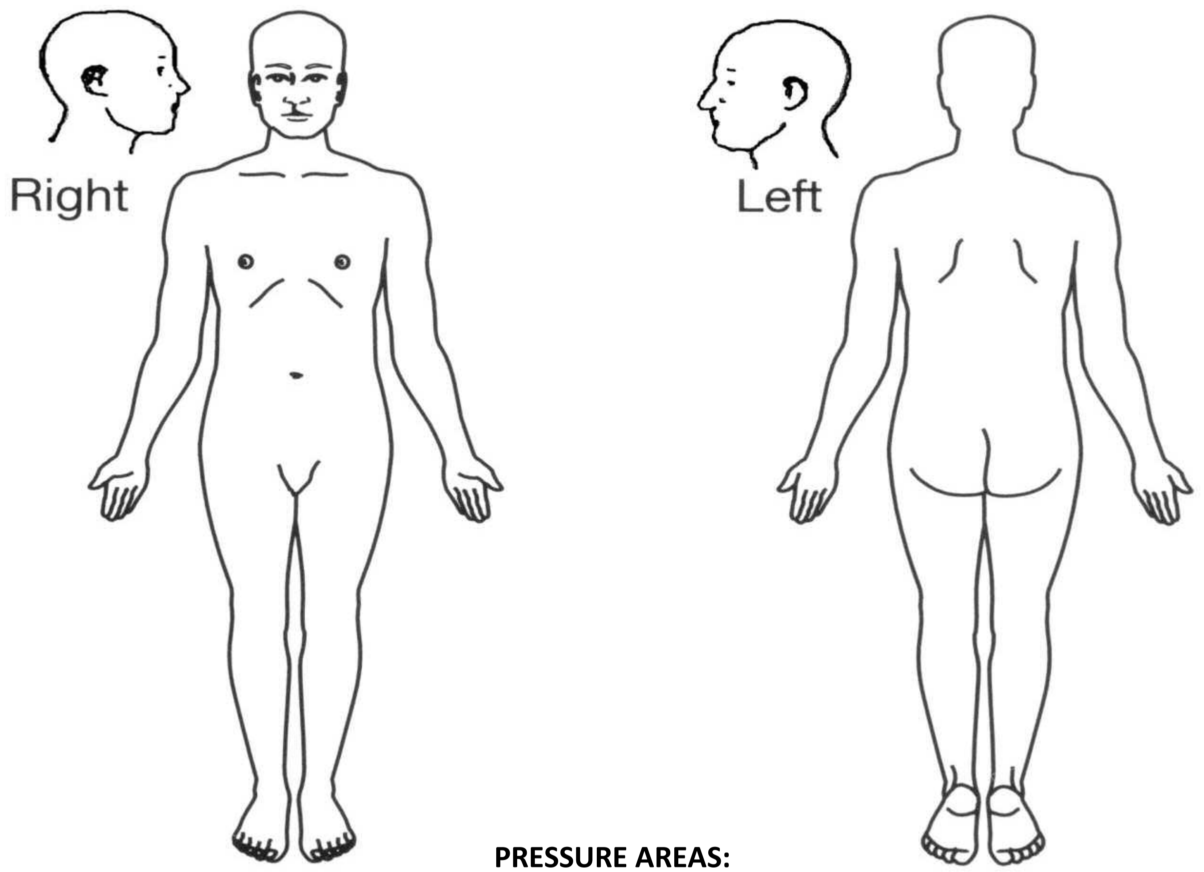

PRESSURE AREAS:

(Complete this for patients with a score of 10 and above as a minimum daily requirement)

Date: →								
**Time: →**								
**Sacrum**								
**Right Buttock**								
**Left Buttock**								
**Natal Cleft**								
**Left Heel**								
**Right Heel**								
**Right Elbow**								
**Left Elbow**								
**Head**								
**Other**								
**Assessors Signature**								

Code:

**N**	Normal
**RB**	Red and Blanching
**RNB**	Red and non Blanching
**PU**	Pressure ulcer (state the grade & document on a wound assessment chart to provide a management plan)
**M**	Moisture/excoriation

*NB Pressure ulcers of grade 2 or above must be reported via CAE during the shift identified.

Risk Assessment Number 8
CONTINENCE

FOR SCANNING PURPOSES
AFFIX PATIENT LABEL

Medical Condition		Does the patient leak urine?		Mental Condition	
Parkinson's Disease	3	Yes	15	Alert	0
Spinal injury	3	No	0	Communicates a little	1
Radical prostatectomy	3	**Activity**		Unable to communicate	2
Physical disability	3	Ambulant	0	Confused	3
Diabetes	3	Limited	1	**Mobility**	
Multiple Sclerosis	3	Very Limited	2	Independent/mobile	0
Dementia	3	Unable to move	3	Mobile with assistance	1
Learning Disability	3	**Bowels**		Transfers bed-chair	2
Post operative surgery	3	Regular/Soft/No assistance	1	Immobile-bedbound	3
**Dexterity**		Regular with laxatives	2	**Medication**	
Good	1	Constipated	3	Diuretics	3
Fair	2	**State the specific changes, if any, in nursing documentation**		Hypnotics	3
Poor	3			Thiazides	3
				Antimuscarinic drugs	3
				Anticholergenic drugs	3
				**If the score is above 14 please continue to answer questions below to identify relevant care plan**	

Date	Score	Signature	

Care Plans Located on Trust Intranet	YES	NO
**Managing Incontinence – patients who are not alert/able to respond to commands/communicate needs**		
Is the patient's conscious level too low to be aware of the need to void?	☐	☐
**If the answer is yes to the above, please refer to care plan 1**		
**Stress Incontinence**		
Do you experience leakage on exertion e.g. laughing, coughing, sneezing, running or jumping?	☐	☐
**If the answer is Yes to the above, please refer to care plan 2**		
**Urge Incontinence**		
Do you experience uncontrollable urge to pass urine?	☐	☐
Do you go to the toilet to pass urine >8 times in 24 hours?	☐	☐
Do you leak large amounts prior to reaching the toilet?	☐	☐
**If the answer if Yes to any of the above, please refer to care plan 3**		
**Output/Overflow Incontinence**		
Do you have difficulty getting the flow started	☐	☐
Do you find the flow slow or erratic?	☐	☐
Do you experience the feeling of incomplete voiding?	☐	☐
Do you suffer with dribbling?	☐	☐
**If the answer is Yes to any of the above, please refer to care plan 4**		
**Functional Incontinence**		
Is the patient confused/disorientated?	☐	☐
Has the patient got mobility problems?	☐	☐
Has the patient got poor vision?	☐	☐
Does the patient have impaired dexterity?	☐	☐
**If the answer is yes to any of the above, please refer to care plan 5**		
**Mixed Urinary Incontinence**		
Leakage on exertion e.g. cough/laugh/sneeze?	☐	☐
Do you have urgency/frequency >8 times in 24 hours?	☐	☐
Do you have Nocturia >2 times at night?	☐	☐
Do you experience uncontrollable urge to pass urine?	☐	☐
**If the answer is yes to any of the above, please refer to care plan 6**		

Risk Assessment Number 7
BEDRAILS

BEDRAILS ASSESSMENT FOR ALL PATIENTS

(See Information for Bedrail Risks Sheet)

DO NOT USE BEDRAILS IF THE FOLLOWING APPLY

- **If the patient has the potential to climb out of bed due to their confused state**
- **To restrain a patient e.g. to keep an agitated patient in bed**
- **As routine where there is no risk of falls**

Section A – Bedrail Risk Assessment

Complete sections A1 and A2, Use your professional judgement to consider the risks and benefits for individual patients. The colour codes should be used as guidance only.

A1- Bedrail Risk Matrix Tool — Circle colour that is most relevant

KEY	
Green	Consider use of bedrails. Risk Assess each shift
Amber	Consider use of bedrails with care. Risk Assess every 4 hours
Red	Bedrails NOT recommended. Risk Assess every 24 hours

Mental state			Mobility 1: Patient is very immobile (bedfast- or hoist-dependant)	Mobility 2: Patient is neither independent nor immobile i.e. requires assistance	Mobility 3: Patient can mobilise without help from staff
	A	Patient is confused, agitated, restless or disorientated	Bedrail may be used with care	Bedrails not recommended	Bedrails not recommended
	B	Patient is drowsy	Bedrails can be used if required	Bedrail may be used with care	Bedrails not recommended
	C	Patient is orientated and alert	Bedrails can be used if required	Bedrails can be used if required	Bedrails not recommended
	D	Patient is unconscious	Bedrails can be used if required	N/A	N/A

DOCUMENT IN NURSING NOTES RATIONALE IF DIFFERENT FROM ABOVE

A2- Alternative Solutions

Consider alternative solutions to bedrails if the risks are greater than the benefits. ✓ Tick all that apply:

Move to observable area to maximise supervision		Crash mat	
Pressure / movement alarm system		Review intentional rounding frequency	
Bed in lowest height after care delivery		Body positioning devices e.g. bed wedges, pillows	
Ultra-low bed		Completion of the 'Forget-Me-Knot' Care Bundle	

Section B–Safer Use of Bedrails

	Tick box
Risk factors discussed with the patient/carer and agreement with the decision made	☐
Staff to refer to bedrail information for patients	☐
Give Bedrail information to Carer and patient	☐

Bedrail Risk Assessment Outcome

Date	Time (24hour)	Assessment Colour and code e.g B3	Recommendation Y/N/Y with Care	Patient and/or Carer Informed	Name/Signature/PIN

Use Bed Rail Continuation Sheet which can be found in the Falls Prevention Booklet

Risk Assessment Number 4
MALNUTRITION UNIVERSAL SCREENING TOOL

FOR SCANNING PURPOSES
AFFIX PATIENT LABEL

Usual / Pre illness weight (kg)……………………………………	Height (m)……………………(actual / recall / ulna)

**Current Weight (Kg)** *If unable to weigh ask patient if recent weight known* →								
**BMI (Enter Value)** →								
NB: If weight & height cannot be obtained, use subjective criteria to inform overall opinion of risk category								
**STEP 1: BMI Score**<br>**Body Mass Index (kg/m²)** (Circle one only)	**<18.5 = Very underweight**	2	2	2	2	2	2	2
	**18.5 – 19.9 = Underweight**	1	1	1	1	1	1	1
	20 – 24.9 = Healthy weight	0	0	0	0	0	0	0
	25-29 = Overweight	0	0	0	0	0	0	0
	>30 (or >28 + Diabetes/ Heart Disease) =Obese	0	0	0	0	0	0	0
**STEP 2: Weight Loss Score**<br>**Unintentional weight loss in last 3 months** (Circle one only)	**> 10% weight loss**	2	2	2	2	2	2	2
	**5 – 10% weight loss**	1	1	1	1	1	1	1
	< 5% weight loss	0	0	0	0	0	0	0
**STEP 3: Acute Disease Effect Score**<br>Examples of acute illness;<br>Acute exacerbation of a chronic disease<br>Cancer<br>Major surgery or Trauma<br>Severe Infection/ Sepsis<br>Severe vomiting<br>Pressure sores Grade 3-4<br>Recent Stroke<br>Chronic diarrhoea/Malabsorption	**YES**<br>**Patient has an acute illness AND there has been or is likely to be no nutritional intake for >5 days**	2	2	2	2	2	2	2
	**NO**	0	0	0	0	0	0	0
	**Total Score**							

Score	Risk	Action	Tick appropriate risk category (Tick one only)						
0	Low	• Repeat screening weekly. If patient 'Obese' consider outpatient referral to Dietitian for weight loss advice.							
1	Medium	• Observe and record food and drink intake (Nutrition Charts / Fluid balance charts)<br>• Highlight risk at nursing handover and on medical rounds<br>• Offer milky drinks and snacks<br>• Encourage high calorie meal choices<br>• Repeat Screening Weekly							
2+	High	• **Inform medical team**<br>• **Refer to Dietitian**<br>• Follow actions for medium risk							
		**Signature →**							
		**Date→**							

REPEAT SCREENING WEEKLY AND DOCUMENT IN NURSING RECORDS

The 'Malnutrition Universal Screening Tool' ('MUST') is adapted / reproduced here with the kind permission of BAPEN (British Association for Parenteral and Enteral Nutrition). For further information on 'MUST' see www.bapen.org.uk

Risk Assessment Number 5
MOVING AND HANDLING

Date: →									
**BMI**									
< 18.5	1	1	1	1	1	1	1	1	1
18.5-20	1	1	1	1	1	1	1	1	1
21-26	0	0	0	0	0	0	0	0	0
27-30	2	2	2	2	2	2	2	2	2
>30	3	3	3	3	3	3	3	3	3
**Mobility/Ability**									
Independent/Fully mobile	0	0	0	0	0	0	0	0	0
Minimal assistance (1 person)	1	1	1	1	1	1	1	1	1
Moderate Assistance (2 people)	2	2	2	2	2	2	2	2	2
Prone to balance loss/Vertigo	3	3	3	3	3	3	3	3	3
Highly Dependent/Immobile	3	3	3	3	3	3	3	3	3
History of falls	3	3	3	3	3	3	3	3	3
Unconscious/semi-conscious	3	3	3	3	3	3	3	3	3
**Communication/Comprehension**									
No difficulty	0	0	0	0	0	0	0	0	0
Deafness	1	1	1	1	1	1	1	1	1
Visual impairment	2	2	2	2	2	2	2	2	2
Language barrier	2	2	2	2	2	2	2	2	2
Confused/Disorientated	3	3	3	3	3	3	3	3	3
**Behavioural constraints**									
Co-operative	0	0	0	0	0	0	0	0	0
Lacks confidence	1	1	1	1	1	1	1	1	1
Poor motivation	1	1	1	1	1	1	1	1	1
Uncooperative	2	2	2	2	2	2	2	2	2
Unpredictable	2	2	2	2	2	2	2	2	2
Aggressive	3	3	3	3	3	3	3	3	3
**Special Factors**									
Equipment in situ	1	1	1	1	1	1	1	1	1
Body Shape (Tall/Short)	1	1	1	1	1	1	1	1	1
Pain/Stiffness	3	3	3	3	3	3	3	3	3
Medications e.g. sedatives	3	3	3	3	3	3	3	3	3
Major surgery/trauma in last 24 hours	4	4	4	4	4	4	4	4	4
**Total Score**									
**Signature of assessor**									

<7 Low Risk | **8 – 14 Medium Risk** | **16> High Risk**

Please use the care plan located via the trust intranet on the front page Left column Click 'CLINICAL SUPPORT', then select 'NURSING DOCUMENTATION'

Risk Assessment Number 6
FALLS

FOR SCANNING PURPOSES
AFFIX PATIENT LABEL

Complete this assessment within 4 HOURS of admission AND on every transfer.
Reassess after a fall, after any relevant change in your patient and also weekly.

**DOES YOUR PATIENT HAVE OR IS YOUR PATIENT?**	Admission Date & Time	Transfer Date & Time	Transfer Date & Time	Transfer Date & Time	Transfer Date & Time
	Ward	Ward	Ward	Ward	Ward
	Signature PIN	Signature PIN	Signature PIN	Signature PIN	Signature PIN
	Print Name	Print Name	Print Name	Print Name	Print Name
	Circle appropriately				
**Aged 65 and over?**	YES/NO	YES/NO	YES/NO	YES/NO	YES/NO

If Yes to the above question please COMPLETE the following:
- **Bedrails assessment Page 8**

Then complete the following documents in the Falls Prevention Booklet
- **Individualised Falls Assessment**
- **Individualised Falls Care Plan**

Reassessment maybe required post-operatively

If the person is 64 and below and answers YES to below please complete the following;
- **Bedrails assessment Page 8**

Then complete the following documents in the Falls Prevention Booklet
- **Individualised Falls Assessment**
- **Individualised Falls Care Plan**

**Is 64 and under with an underlying condition? e.g. Stroke, Dementia, Parkinson's**	YES/NO	YES/NO	YES/NO	YES/NO	YES/NO
**Acute confusion (delirium)?**	YES/NO	YES/NO	YES/NO	YES/NO	YES/NO
**Dementia?**	YES/NO	YES/NO	YES/NO	YES/NO	YES/NO
**Agitation?**	YES/NO	YES/NO	YES/NO	YES/NO	YES/NO
**Drowsy?**	YES/NO	YES/NO	YES/NO	YES/NO	YES/NO
**History of falls within last month?**	YES/NO	YES/NO	YES/NO	YES/NO	YES/NO
**Admitted with a fall?**	YES/NO	YES/NO	YES/NO	YES/NO	YES/NO
**Suffered a fall during hospital stay?**	YES/NO	YES/NO	YES/NO	YES/NO	YES/NO
**Unwilling/unable to use the call bell system?**	YES/NO	YES/NO	YES/NO	YES/NO	YES/NO
**Unsteady on their feet?**	YES/NO	YES/NO	YES/NO	YES/NO	YES/NO
**Unsafe or trying to get up unaided?**	YES/NO	YES/NO	YES/NO	YES/NO	YES/NO
**History of alcohol/drugs?**	YES/NO	YES/NO	YES/NO	YES/NO	YES/NO
**Under the influence alcohol/drugs?**	YES/NO	YES/NO	YES/NO	YES/NO	YES/NO
**Withdrawing from alcohol/drugs?**	YES/NO	YES/NO	YES/NO	YES/NO	YES/NO

Any other reason you feel your patient may be vulnerable to falling? Outline please

If you have answered YES to any of the above questions please complete the relevant sections of the 'Individualised Falls Assessment and Care Plan' in the Falls Prevention Booklet once the bedrails assessment is completed

Appendix A

POST FALL PROTOCOL

If your patient has a fall complete all of the following

SAFETY STEPS	SIGN AND DATE/TIME IN EACH BOX			
	Fall No1<br>DATE/TIME	Fall No2<br>DATE/TIME	Fall No3<br>DATE/TIME	Fall No4<br>DATE/TIME
**Environment** Check the environment for hazards	Signature	Signature	Signature	Signature
**Pain** Ask the patient 'are they experiencing any pain'. **If yes**<br>• Ask to indicate where the pain is located.<br>• Visually check patient's body for any sign of bony injury, cuts, swelling or bruising.<br>• **Offer pain relief**	Signature	Signature	Signature	Signature
**Observations**<br>• Take vital signs<br>• Check level of consciousness<br>• **If head injury is suspected** perform neurological observations as per protocol (NICE Guideline 76). Refer to 'Suspected Head Injury Neurological Observations Protocol'- Intranet Falls Prevention	Signature	Signature	Signature	Signature
**If serious injury** is **suspected** (fracture, spinal injury and head injury)<br>• Make the patient comfortable **and await medical review before moving the patient.**<br>• When contacting Dr request review within 30 minutes (as per NICE guideline) and keep the patient NBM until then.	Signature	Signature	Signature	Signature
If serious injury is **confirmed** by Dr then the patient must be:<br>• Transferred to bed using the **Molift hoist and scoop stretcher** (**UHCW** located in Wd 22 or Equipment Library, **Rugby** Mulberry Ward, hoist with the scoop).<br>• Seek advice from the handling and moving team if unsure how to use this equipment.	Signature	Signature	Signature	Signature
If serious injury is **not suspected** either/or<br>• Guide patient from the floor using the "**backward chaining**" method (in falls resource folder and intranet)<br>• If patient has mobility problems use appropriate **hoist** to move the patient back into bed. Inform Dr and request review within 12 hours (as per NICE guidelines). Place medical review sticker in the notes for Dr Review	Signature	Signature	Signature	Signature
**Complete a detailed Incident Report on Datix**, consider if incident should be reported to RIDDOR<br>**Ensure Duty of Candour** is started by informing, patient, relatives or carer of the incident. Document **who** has informed NOK and to **whom**, with the date and time of notification.<br>**Document detail in Nursing notes including how the patient was moved from the floor**	Signature	Signature	Signature	Signature
Ensure Dr completes **'Medical Report after a fall'** document which is to be inserted in patient notes.<br>Review falls care-plan, risk factors, review medications Bed Rails Assessment Implement any relevant actions<br>Perform lying and standing blood pressure as soon as condition allows.	Signature	Signature	Signature	Signature
CHECK if **X-ray/Radiology** has been;<br>Requested<br>Taken<br>Fracture confirmed<br>Results reported	Yes ☐ No ☐<br>Yes ☐ No ☐<br>Yes ☐ No ☐<br>Yes ☐ No ☐<br>Signature	Yes ☐ No ☐<br>Yes ☐ No ☐<br>Yes ☐ No ☐<br>Yes ☐ No ☐<br>Signature	Yes ☐ No ☐<br>Yes ☐ No ☐<br>Yes ☐ No ☐<br>Yes ☐ No ☐<br>Signature	Yes ☐ No ☐<br>Yes ☐ No ☐<br>Yes ☐ No ☐<br>Yes ☐ No ☐<br>Signature
Complete a **Post Falls Group Reflection** document keeping copies on the Ward AND in the Medical Notes	Signature	Signature	Signature	Signature

NHS
University Hospitals
Coventry and Warwickshire
NHS Trust

Falls Prevention Booklet

Individualised Falls Assessment and Care Plan,
Post Falls Documentation and
Bedrail Continuation Sheet

Complete the following documents for ***every patient*** *who has been identified as being at* ***risk of falling*** *using the patient risk assessment booklet*

FOR SCANNING PURPOSES
AFFIX PATIENT LABEL

Process for Completing this booklet

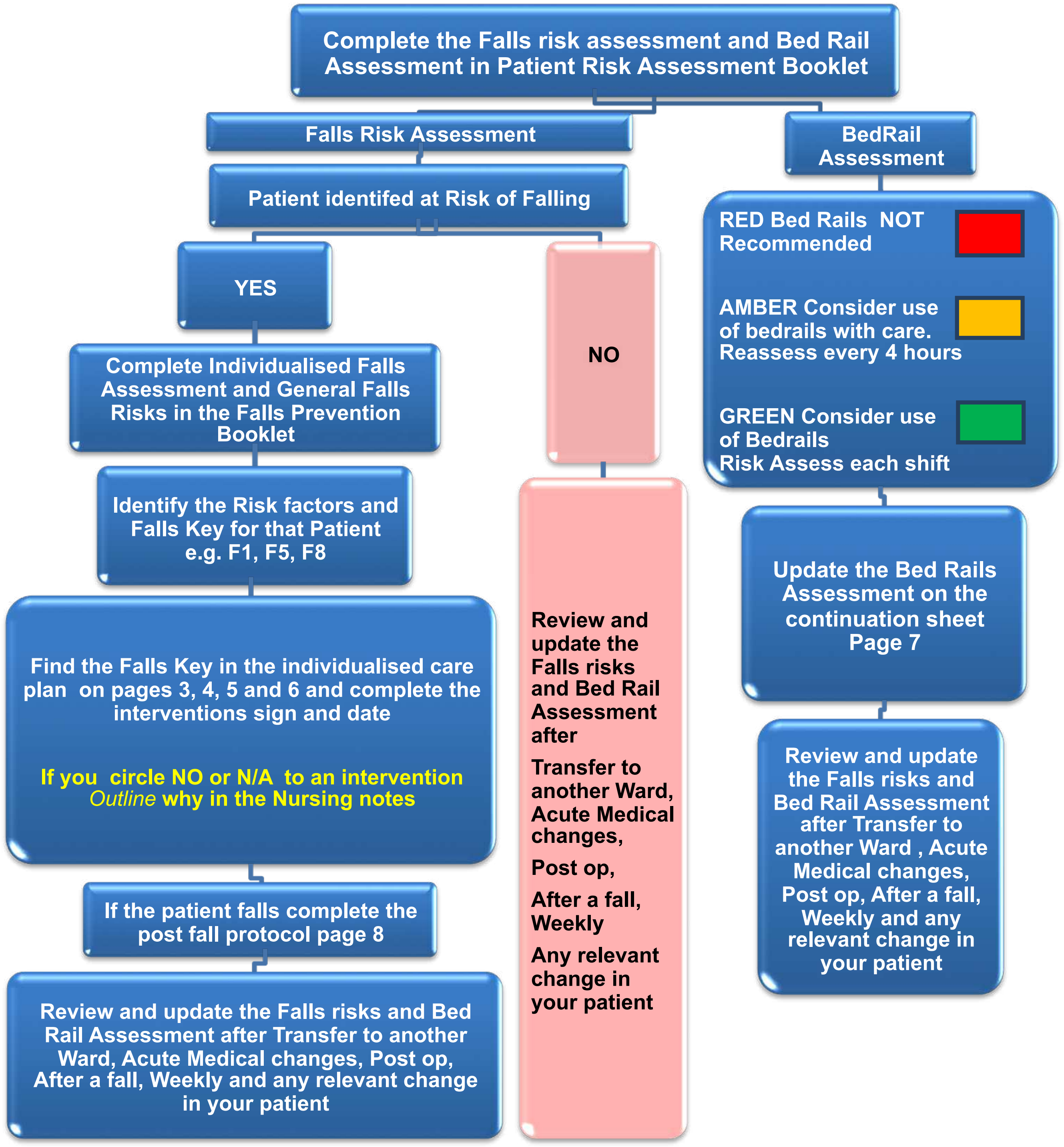

Together Towards World Class

Individualised Falls Assessment

Complete after Transfer to another Ward; Weekly; Acute medical changes; Post op; After a fall; or after any other relevant change in your patient.

Individual Multifactorial Risk Factors

Falls KEY	Risk Factors	YES/NO	YES/NO	YES/NO	YES/NO
**F1**	History of falls, syncope, dizziness, collapse, postural hypotension				
**F2**	Polypharmacy or prescribed medications that increase falls risk				
**F3**	Hearing or visual impairment				
**F4**	Acute confusion				
**F5**	Unfamiliar Surroundings/Orientation difficulties				
**F6**	Urinary and/or bowel symptoms				
**F7**	Risk of dehydration and/or malnourishment				
**F8**	Fear of falling / lack of awareness of falls risks				
**F9**	Change in condition and/or comorbidities predisposing pt to a fall				
**F10**	Concerns re balance, mobility, gait, wheelchair or mobility aids				
**F11**	Cognitive impairment / Dementia				
**F12**	Unable or unwilling to use the call bell				
**F13**	Post anaesthesia within 24 hours or on post op analgesia				

Once the patient's risk factors have been identified above, complete the general falls risk factors below. After this complete the individualised falls care plan relating to the identified risks above. ONLY complete the care-plan corresponding to relevant identified sections relating to the key on the left e.g. F8, F11

General Falls Risks	ADMISSION	REVIEW	REVIEW	REVIEW
**Safety & Communication: Tick when completed sign and date**				
Discuss with patient and Carers their risks for falling				
Ensure call bell explained and within easy reach if able to use (prevent over reaching)				
Patient can reach their belongings safely				
Falling Stars are in place as appropriate				
**Equipment: Assess the need for bedrails**				
If likely to fall from bed ensure bed at lowest height unless this reduces mobility or independence				
**Location on Ward: Consider the patients' location on the ward/area**				
Select appropriate place when considering patient's needs e.g. close to nurse's station, near to a toilet, quiet area or away from the door. Consider lighting best for patient e.g. bedside light left on overnight				
**Safe Footwear: Check footwear for secure fit - non slip sole or trailing laces**				
Provide and explain red non-slip socks- contact relatives to provide safe footwear				
**Level of Observation:**				
Ensure 'Intentional rounding tool' is completed<br>Consider and decide on level of observation e.g. General, Cohort, or 'Enhanced Care'.<br>Outline in Nursing Notes observation level required.				
	Date	Date	Date	Date
	Time	Time	Time	Time
	Ward	Ward	Ward	Ward
	Signature PIN	Signature PIN	Signature PIN	Signature PIN

FOR SCANNING PURPOSES
AFFIX PATIENT LABEL

Bedrail Risk Assessment Continuation Sheet

A1- Bedrail Risk Matrix Tool Identify the risk colour that is most relevant and document below

KEY		
Green	Consider use of bedrails. Risk Assess each shift	
Amber	Consider use of bedrails with care. Risk Assess every 4 hours	
Red	Bedrails NOT recommended. Risk Assess every 24 hours	

Mental state		Mobility 1: Patient is very immobile (bedfast- or hoist-dependant)	Mobility 2: Patient is neither independent nor immobile i.e. requires assistance	Mobility 3: Patient can mobilise without help from staff
A	Patient is confused, agitated, restless or disorientated	Bedrail may be used with care	Bedrails not recommended	Bedrails not recommended
B	Patient is drowsy	Bedrails can be used if required	Bedrail may be used with care	Bedrails not recommended
C	Patient is orientated and alert	Bedrails can be used if required	Bedrails can be used if required	Bedrails not recommended
D	Patient is unconscious	Bedrails can be used if required	N/A	N/A

DOCUMENT IN NURSING NOTES RATIONALE IF DIFFERENT FROM ABOVE

Date	Time (24hour)	Assessment Colour and code e.g. RED B3	Recommendation Y/N/Y with Care	Patient and/or Carer Informed	Name/Signature/PIN

F11	Cognitive impairment / Dementia	• Implement dementia care bundle, including Getting to know you form, blue pillow case, regular communication to offer reassurance and explanation, or written prompts/communication aids	Commence Forget-me Not' care bundle	Y / N / NA	Y / N / NA	Y / N / NA
			Requires intentional rounding and handover	Y / N / NA	Y / N / NA	Y / N / NA
			Blue pillow case insitu	Y / N / NA	Y / N / NA	Y / N / NA
			Regular communication to provide explanation and reassurance	Y / N / NA	Y / N / NA	Y / N / NA
			Request relatives / carers bring in familiar items	Y / N / NA	Y / N / NA	Y / N / NA
F12	Unable or unwilling to use the call bell	• Reason why patient unable to use call bell system to be determined i.e functional or cognitive<br>• Nurse in a visible bed space in order to maintain patient safety<br>• Ensure all members of multidisciplinary team are aware patient unable / unwilling to utilise the call bell system<br>• Ward staff to consider alternative call bells and anticipate patient need<br>• Consider use of falls alarm but this may not be appropriate for an agitated patient	Intentional rounding to be completed hourly	Y / N / NA	Y / N / NA	Y / N / NA
			Inability to utilise call bell documented in nursing notes and on CRRS /e-handover	Y / N / NA	Y / N / NA	Y / N / NA
			Nursed in visible position	Y / N / NA	Y / N / NA	Y / N / NA
			Falls alarm in use	Y / N / NA	Y / N / NA	Y / N / NA
			Relatives informed of possible increase in falls	Y / N / NA	Y / N / NA	Y / N / NA
			Alternative call bell systems in use	Y / N / NA	Y / N / NA	Y / N / NA
F13	Post anaesthesia within 24 hours or on post op analgesia	• Consider impact of anaesthetic, surgical procedure and analgesia on mobility and risk of falls<br>• Identify mobility risks i.e. surgical drains, IVI, urinary catheter, feeding tubes, PCA<br>• Offer clear concise instructions re safety when mobilising post operatively or whilst on analgesia<br>• Ensure patient understanding of risks identified<br>• Call bell system explained and within easy reach<br>• If patient reports dizziness perform lying and standing BP, document and inform surgical team of result	Communicate tailored moving and handling from Medical Team post-op	Y / N / NA	Y / N / NA	Y / N / NA
			Mobility risk explained to patient	Y / N / NA	Y / N / NA	Y / N / NA
			Ensure placement of drains, drips catheters i.e. drain and catheter are NOT positioned on different sides of the bed. Ensure mobility is not impeded.	Y / N / NA	Y / N / NA	Y / N / NA
			Check footwear to ensure secure & non-slip	Y / N / NA	Y / N / NA	Y / N / NA
			Ensure appropriate mobility aids provided and within easy reach	Y / N / NA	Y / N / NA	Y / N / NA
			Referral made to PT / OT	Y / N / NA	Y / N / NA	Y / N / NA
			Call bell and belongings are within easy reach	Y / N / NA	Y / N / NA	Y / N / NA
				**Signature PIN**	**Signature PIN**	**Signature PIN**

FOR SCANNING PURPOSES AFFIX PATIENT LABEL

INDIVIDUALISED FALLS CARE PLAN

It is not necessary to repeat identical interventions. Refer back to the already completed risk section
Communicate the interventions to the relevant Team
Document why your answer is YES or NO in the Nursing notes

KEY	Risk	Care Plan	Intervention; *Enter results into notes and discuss with medical team*	Date/Time *Circle*	Date/Time *Circle*	Date/Time *Circle*
F1	History of falls, syncope, dizziness, collapse, postural hypotension	• Monitor Lying and Standing BP & inform medical team of result. Record X3 as per guidance. Record deficit noted in Vital Pac<br>• Educate patient to get up slowly when sit to standing<br>• Perform ECG if instructed by medical team<br>• Offer assistance when mobilising<br>• Refer as appropriate to PT<br>• Consider use of ultralow beds<br>• Explain call bell system	1. Lying/Standing record result in Vital Pac	Y / N / NA	Y / N / NA	Y / N / NA
			2. Lying/Standing record result in Vital Pac	Y / N / NA	Y / N / NA	Y / N / NA
			3. Lying/Standing record result in Vital Pac	Y / N / NA	Y / N / NA	Y / N / NA
			Urinalysis Result and discuss with (D/W) Medical team	Y / N / NA	Y / N / NA	Y / N / NA
			MSU/CSU D/W Medical team	Y / N / NA	Y / N / NA	Y / N / NA
			ECG and D/W Medical team	Y / N / NA	Y / N / NA	Y / N / NA
			Referral made to Physiotherapy (PT)	Y / N / NA	Y / N / NA	Y / N / NA
			Patient Nursed on ultralow bed	Y / N / NA	Y / N / NA	Y / N / NA
			Call bell system explained	Unable to use/ Y/N	Unable to use/ Y/N	Unable to use/ Y/N
F2	Polypharmacy or prescribed medications that increase falls risk	• Review of drug chart by ward pharmacist<br>• Avoid night sedation where possible<br>• Pharmacist to document in medical notes outcome of review<br>• Ensure medications that affect mobility, i.e., Parkinsons Drugs medications, are given as per recommended regime	Request Pharmacy Review of drug kardex regarding falls risks on admission, acute change of condition and/or post fall. ***Identify on E-Hand-over if the patient requires pharmacy review*** (Refer to 'drugs that may increase the falls risk' sheet)	Y / N / NA	Y / N / NA	Y / N / NA
F3	Hearing or visual impairment	• Ensure patient has appropriate aids for hearing and vision<br>• Refer to audiology if concerns re hearing aids<br>• Ensure bed space clutter free<br>• Consider optician follow up	Request relatives/carers to bring in visual or hearing aids	Y / N / NA	Y / N / NA	Y / N / NA
			Document in nursing notes which aids have been left with patient	Y / N / NA	Y / N / NA	Y / N / NA
			Referral to audiology	Y / N / NA	Y / N / NA	Y / N / NA
			Advise to follow up with Optician	Y / N / NA	Y / N / NA	Y / N / NA

**F4**	**Acute confusion - consider: -**<br>• ***Delirium***<br>• ***Sepsis***<br>• ***Head Injury***<br>• ***Alcohol/Drugs***<br>• ***Electrolyte Imbalance***	• Consider use of falls alarm to minimise falls risk ***(falls alarms may be inappropriate for agitated patients)***<br>• Consider need for increased observation i.e. cohort / 1:1<br>• Consider need for DOLS application if increased observation required<br>• Document evidence of challenging behaviour<br>• Consider use of ultralow beds<br>• Ensure call bell in easy reach	Intentional rounding chart in use	**Y / N / NA**	**Y / N / NA**	**Y / N / NA**
			Hourly behaviour charts in use	**Y / N / NA**	**Y / N / NA**	**Y / N / NA**
			Consider using 'getting to know me' form	**Y / N / NA**	**Y / N / NA**	**Y / N / NA**
			Nursed in;	**Cohort bay Y/N 1:1 Y/N**	**Cohort bay Y/N 1:1 Y/N**	**Cohort bay Y/N 1:1 Y/N**
			Call bell system explained , within reach	**Y /N/Unable to use**	**Y /N/Unable to use**	**Y /N/Unable to use**
			Patient nursed on ultralow bed	**Y / N / NA**	**Y / N / NA**	**Y / N / NA**
			Falls alarm in use	**Y / N / NA**	**Y / N / NA**	**Y / N / NA**
			Referral to Enhanced Care Team	**Y / N / NA**	**Y / N / NA**	**Y / N / NA**
			Deprivation of Liberty Safeguards (DOLS) in place	**Y / N / NA**	**Y / N / NA**	**Y / N / NA**
**F5**	**Unfamiliar Surroundings/ Orientation difficulties**	• Ensure ward layout and routines explained to patient<br>• Ensure patient belongings stored appropriately to reduce falls risk. Advise family re essential items<br>• Consider use of ultralow beds	Orientate patient to ward including bathrooms	**Y / N / NA**	**Y / N / NA**	**Y / N / NA**
			Call bell system explained , within reach	**Y /N/Unable to use**	**Y /N/Unable to use**	**Y /N/Unable to use**
			Patient nursed on ultralow bed	**Y / N / NA**	**Y / N / NA**	**Y / N / NA**
			Bed space kept clutter free	**Y / N / NA**	**Y / N / NA**	**Y / N / NA**
**F6**	**Urinary and bowel symptoms consider:**<br>• ***Urinary incontinence***<br>• ***Urinary frequency, including nocturnal***<br>• ***Pain / Dysuria***<br>• ***Faecal incontinence***<br>• ***Diarrhoea***<br>• ***Constipation***	• Identify evidence of urinary infection (frequency, dysuria and tenderness)<br>• Complete continence assessment<br>• Ensure appropriate use of continence aids<br>• Monitor bowels and manage / treat constipation / diarrhoea<br>• Ensure patient familiar with location of the bathroom<br>• Ensure call bell within easy reach<br>• Consider need for increased observation in bathrooms<br>• Consider need for continence referral on discharge	Urinalysis Result and D/W Medical team	**Y / N / NA**	**Y / N / NA**	**Y / N / NA**
			MSU/CSU D/W Medical team	**Y / N / NA**	**Y / N / NA**	**Y / N / NA**
			Continence risk assessment complete and appropriate care plan in place	**Y / N / NA**	**Y / N / NA**	**Y / N / NA**
			Document chosen continence aids in nursing notes	**Y / N / NA**	**Y / N / NA**	**Y / N / NA**
			Complete daily stool chart	**Y / N / NA**	**Y / N / NA**	**Y / N / NA**
			Administer bowel medication as prescribed	**Y / N / NA**	**Y / N / NA**	**Y / N / NA**
			Orientate patient to bathrooms	**Y /N/Unable to use**	**Y /N/Unable to use**	**Y /N/Unable to use**
			Call bell system explained , within reach	**Y /N/Unable to use**	**Y /N/Unable to use**	**Y /N/Unable to use**
			**Document need for nursing presence in bathroom and update on e-handover**	**Y / N / NA**	**Y / N / NA**	**Y / N / NA**
**F7**	**Risk of dehydration and/or malnourishment**	• Monitor fluid and dietary intake<br>• Explore patients fluid and dietary preferences<br>• Offer assistance with meals and drinks to optimise hydration and nutritional intake as required	Complete MUST assessment including weight and BMI	**Y / N / NA**	**Y / N / NA**	**Y / N / NA**
			Complete fluid balance / hydration, nutrition charts and intentional rounding charts	**Y / N / NA**	**Y / N / NA**	**Y / N / NA**

FOR SCANNING PURPOSES
AFFIX PATIENT LABEL

**F8**	**Fear of falling / lack of awareness of falls risks**	• Reduce patient/carers anxiety / increase awareness re falls risks by providing falls information leaflet<br>• Explore/discuss with patients their fear of falling<br>• Inform multidisciplinary team of patients anxiety re falls	Falls leaflet provided	**Y / N / NA**	**Y / N / NA**	**Y / N / NA**
			Referred to PT / Occupational Therapy (OT)	**Y / N / NA**	**Y / N / NA**	**Y / N / NA**
**F9**	**Change in condition and or comorbidities predisposing patients to fall, i.e,**<br>• ***Sepsis***<br>• ***Parkinson's disease***<br>• ***Diabetes***<br>• ***Postural hypotension***<br>• ***Stroke***<br>• ***Frailty***<br>• ***Osteoporosis***	• Monitor NEWS as per protocol and escalate any acute changes to medical team using SBAR<br>• Acknowledge the effects of illness on the risk of falls	Ensure NEWS is recorded and escalated in line with Trust Policy	**Y / N / NA**	**Y / N / NA**	**Y / N / NA**
			Monitor for signs of sepsis, i.e urinary frequency, cough etc And escalate changes to MDT.	**Y / N / NA**	**Y / N / NA**	**Y / N / NA**
			Communicate specific medication regimes i.e, PD meds are given as prescribed	**Y / N / NA**	**Y / N / NA**	**Y / N / NA**
			Monitor, record and escalate blood sugars if abnormal	**Y / N / NA**	**Y / N / NA**	**Y / N / NA**
			Refer to specialist nurses for management advice	**Y / N / NA**	**Y / N / NA**	**Y / N / NA**
			Referral made to PT / OT	**Y / N / NA**	**Y / N / NA**	**Y / N / NA**
**F10**	**Concerns re balance, mobility, gait, wheelchair or mobility aids**	• Identify mobility risks i.e. surgical drains, IVI, urinary catheter, feeding tubes, PCA<br>• Assess mobility and provide assistance/equipment were required<br>• If dizziness noted record lying and standing BP in Vital Pac<br>• Consider use of falls alarm however this may not be appropriate for an agitated patient<br>• Consider need for increased observation<br>• Consider use of ultralow bed<br>• Referral to PT/OT for assessment<br>• Ensure call bell and belongings are within easy reach	Complete handling and moving assessment and appropriate care plan	**Y / N / NA**	**Y / N / NA**	**Y / N / NA**
			Lying/Standing BP record result in Vital Pac	**Y / N / NA**	**Y / N / NA**	**Y / N / NA**
			Check footwear to ensure secure & non-slip	**Y / N / NA**	**Y / N / NA**	**Y / N / NA**
			Ensure appropriate mobility aids provided and within easy reach	**Y / N / NA**	**Y / N / NA**	**Y / N / NA**
			Referral made to PT / OT	**Y / N / NA**	**Y / N / NA**	**Y / N / NA**
			Patient provided with non-slip socks	**Y / N / NA**	**Y / N / NA**	**Y / N / NA**
			Call bell and belongings are within easy reach	**Y / N / NA**	**Y / N / NA**	**Y / N / NA**
			Falls alarm in use	**Y / N / NA**	**Y / N / NA**	**Y / N / NA**
			Increased observation required	**Y / N / NA**	**Y / N / NA**	**Y / N / NA**
			Nursed on ultralow bed	**Y / N / NA**	**Y / N / NA**	**Y / N / NA**
