# Supplementary material for: An opportunistic evaluation of a routine service improvement project to reduce falls in hospital
Source: BMC Health Serv Res. 2021 Jan 22;21:79. doi: 10.1186/s12913-021-06073-4 (PMC7821491; doi:10.1186/s12913-021-06073-4)
Supplement: Supplementary file 2 — Additional file 2: Appendix B. Supplementary tables and figures. [file 12913_2021_6073_MOESM2_ESM.pdf]

Appendix B. Supplementary tables and figures

Appendix B, Table S1: Evaluation of intervention using cubic splines for pre- and post-intervention time.

Number of falls per 1000 bed days		Intervention	
Control	Intervention	IRR (95% CI)	p-value
6.62	5.89	4.34 (0.61 to 31.10)	0.144

IRR: Incidence rate ratio. CI: Confidence interval

Appendix B, Table S2: Model parameters for cubic spline model

Parameter	IRR	95% CI	p-value
**Intervention exposure**	4.34	0.61 to 31.10	0.144
**Pre intervention spline point 1**	1.06	1.03 to 1.09	0.000
**Pre intervention spline point 2**	0.74	0.62 to 0.88	0.001
**Pre intervention spline point 3**	1.89	1.19 to 3.02	0.007
**Pre intervention spline point 4**	0.67	0.36 to 1.25	0.204
**Post intervention spline point 1**	0.95	0.87 to 1.02	0.165
**Post intervention spline point 2**	0.97	0.66 to 1.44	0.892
**Post intervention spline point 3**	1.46	0.27 to 7.96	0.663
**Post intervention spline point 4**	0.51	0.05 to 5.10	0.566
**Initial rate of falls**	5.42	4.66 to 6.30	

IRR: Incidence rate ratio. CI: Confidence interval. Model was fitted with a fixed effect for intervention, a cubic spline for time pre-intervention, and a cubic spline for time post-intervention.

Appendix B, Figure S1: Partial autocorrelation plots for individual wards

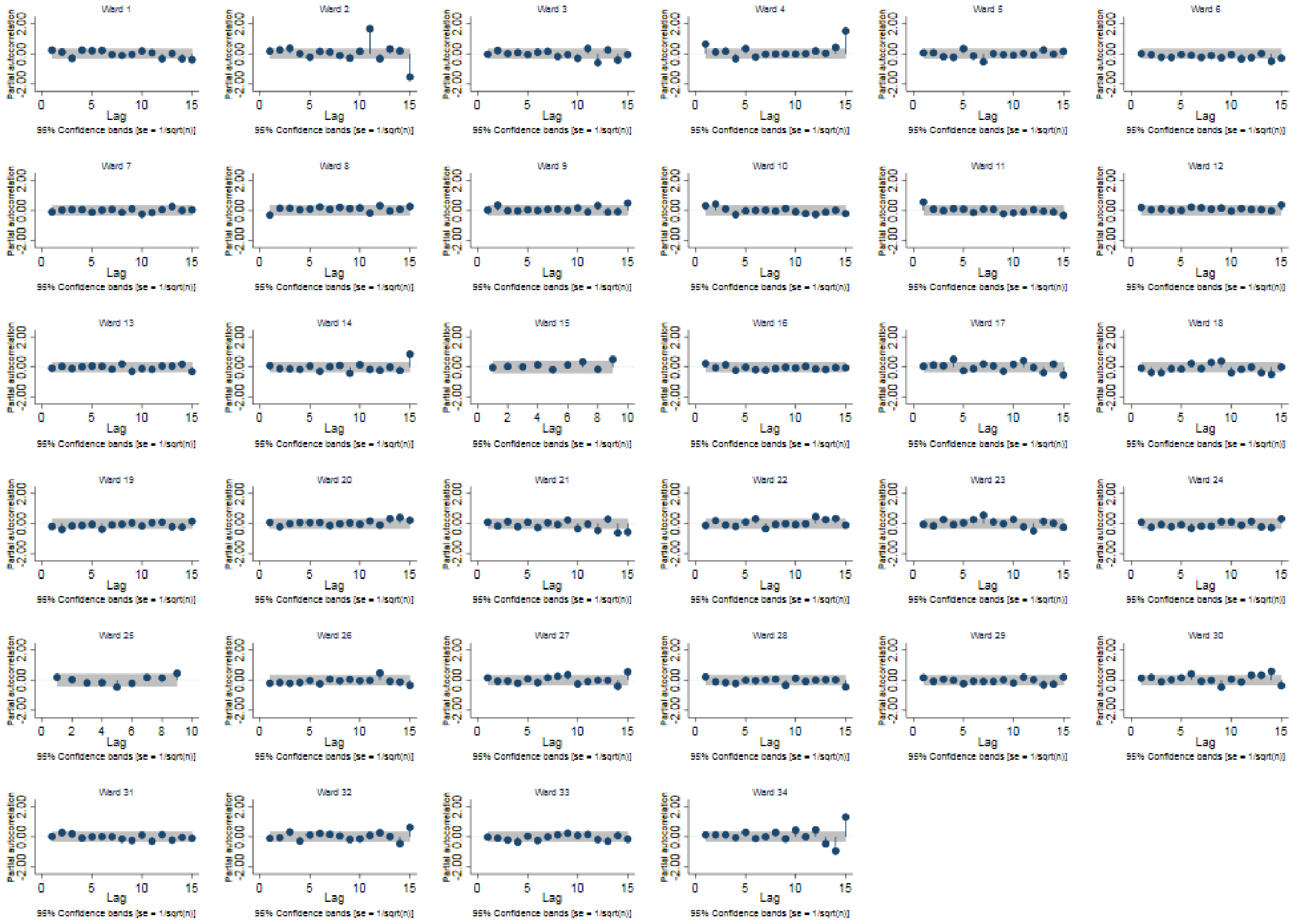

Appendix B, Figure S2: Observed vs predicted falls per 1000 bed days

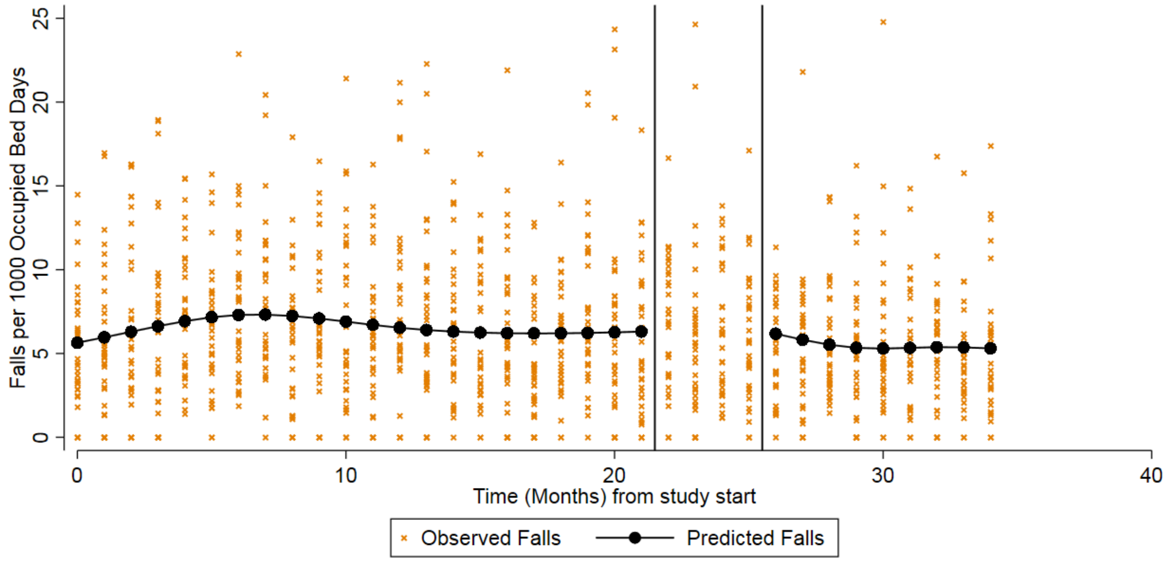

The black vertical lines indicate the beginning and end of the intervention implementation. A mixed effect Poisson model was used to estimate the predicted falls, with a random effect for ward and a cubic spline for pre- and post-intervention time trend.
